# The impact of inter-observer variation in delineation on robustness of radiomics features in non-small cell lung cancer

**DOI:** 10.1038/s41598-022-16520-9

**Published:** 2022-07-27

**Authors:** Gargi Kothari, Beverley Woon, Cameron J. Patrick, James Korte, Leonard Wee, Gerard G. Hanna, Tomas Kron, Nicholas Hardcastle, Shankar Siva

**Affiliations:** 1 Department of Radiation Oncology, Peter MacCallum Cancer Centre, Victorian Comprehensive Cancer Centre Building, 305 Grattan Street, Melbourne, VIC 3000 Australia; 2 Present Address: Sir Peter MacCallum Department of Oncology, Peter MacCallum Cancer Centre, University of Melbourne, Melbourne, VIC Australia; 3 Department of Radiology, Cancer Imaging, Peter MacCallum Cancer Centre, Melbourne, VIC Australia; 4 Statistical Consulting Centre, University of Melbourne, Parkville, Australia; 5 Department of Physical Sciences, Peter MacCallum Cancer Centre, Melbourne, VIC Australia; 6 Department of Biomedical Engineering, School of Chemical and Biomedical Engineering, University of Melbourne, Melbourne, VIC Australia; 7 Department of Radiotherapy (MAASTRO), GROW School of Oncology, Maastricht University Medical Centre+, Maastricht, The Netherlands; 8 Clinical Data Science, Maastricht University, Maastricht, The Netherlands; 9 Centre for Medical Radiation Physics, University of Wollongong, Wollongong, NSW Australia

**Keywords:** Cancer, Cancer imaging, Lung cancer

## Abstract

Artificial intelligence and radiomics have the potential to revolutionise cancer prognostication and personalised treatment. Manual outlining of the tumour volume for extraction of radiomics features (RF) is a subjective process. This study investigates robustness of RF to inter-observer variation (IOV) in contouring in lung cancer. We utilised two public imaging datasets: ‘NSCLC-Radiomics’ and ‘NSCLC-Radiomics-Interobserver1’ (‘Interobserver’). For ‘NSCLC-Radiomics’, we created an additional set of manual contours for 92 patients, and for ‘Interobserver’, there were five manual and five semi-automated contours available for 20 patients. Dice coefficients (DC) were calculated for contours. 1113 RF were extracted including shape, first order and texture features. Intraclass correlation coefficient (ICC) was computed to assess robustness of RF to IOV. Cox regression analysis for overall survival (OS) was performed with a previously published radiomics signature. The median DC ranged from 0.81 (‘NSCLC-Radiomics’) to 0.85 (‘Interobserver’—semi-automated). The median ICC for the ‘NSCLC-Radiomics’, ‘Interobserver’ (manual) and ‘Interobserver’ (semi-automated) were 0.90, 0.88 and 0.93 respectively. The ICC varied by feature type and was lower for first order and gray level co-occurrence matrix (GLCM) features. Shape features had a lower median ICC in the ‘NSCLC-Radiomics’ dataset compared to the ‘Interobserver’ dataset. Survival analysis showed similar separation of curves for three of four RF apart from ‘original_shape_Compactness2’, a feature with low ICC (0.61). The majority of RF are robust to IOV, with first order, GLCM and shape features being the least robust. Semi-automated contouring improves feature stability. Decreased robustness of a feature is significant as it may impact upon the features’ prognostic capability.

## Introduction

Personalising treatment and accurate prognostication are driving factors for advances in medicine. Radiomics has been proposed as a potential tool to advance this. There are several published studies assessing the value of a radiomics based approach in predicting outcomes for oncology patients. These studies found radiomic features to be associated with oncological outcomes including local control and overall survival^1–3^, risk of lymph node metastases^4^, rates of pathological complete response^5^, and tissue biomarkers^6^. Aerts et al. 2014 published a radiomics signature (then reproduced in distributed learning fashion by Shi et al. 2019) that incorporated four radiomics features found to be prognostic in non-small cell lung cancer (NSCLC)^7,8^. Despite the burgeoning literature on prognostic radiomics models, there are ongoing issues with reproducibility of models, with minimal overlap in the radiomics features found to be prognostic between studies^1,9,10^. A number of different factors have been found within the radiomics literature to affect the robustness of radiomics features, including the feature extraction process^11^ and image acquisition parameters such as slice thickness^12^, image reconstruction^13^, kernels^14^, tube voltage and current^15^ and type of scanner^16^. An essential yet challenging part of the current radiomics workflow involves delineation of a volume of interest (VOI). In most cases, delineation remains a process requiring either complete or partial manual effort by an expert. An inherent limitation of this approach is the inter-observer variation (IOV) that this introduces to the radiomics process. Numerous studies have documented that IOV exists in contouring radiation oncology target volumes in different tumour streams^17,18^. Only a few studies have investigated how variation in contouring may affect radiomic feature results. Whether certain radiomics features or feature classes are more or less robust to IOV in contouring is poorly understood, with studies investigating this limited by mixed tumour cohorts, use of non-standardised radiomics features, and/or small patient numbers, and have conflicting findings^19–25^. One study by Haarburger et al. 2020 showed that in patients with lung, liver or kidney lesions, shape and first order radiomics features were the most stable to IOV in delineation^24^, while another study by Pavic et al. 2018 in NSCLC, mesothelioma and head and neck cancer showed that while robustness of radiomics features to IOV in delineation significantly varied by tumour type, shape features were the least stable across all tumour types^22^. In addition to a lack of consistency regarding which radiomics features are most robust to IOV in delineation, there is limited data available regarding the impact this has on the prognostic ability of a radiomics feature. One study by Vuong et al. 2020 showed that choosing only features robust to variation in CT acquisition parameters and IOV in delineation in a multi-centric NSCLC imaging dataset resulted in a prognostic model that performed equally well as a model derived from an imaging dataset with standardised imaging parameters, albeit with different radiomics features included within the models^26^. No studies to our knowledge in NSCLC have specifically investigated whether the use of a different set of contours in patients with NSCLC undergoing radiotherapy will result in the loss of prognostic ability of radiomics features, and whether this is related to the robustness of this feature to IOV in delineation. This large multi-centre study therefore aims to assess the robustness of radiomics features to IOV in contouring and the resultant impact of this on the prognostic power of radiomics features in a homogenous cohort of patients with NSCLC undergoing curative intent radiotherapy.

## Methods

### Datasets

For this study, we utilised de-identified imaging datasets and clinical data from an openly accessible online centralised repository, The Cancer Imaging Archive (TCIA) (https://www.cancerimagingarchive.net)^27^.

The first dataset we employed is a NSCLC cohort from Netherlands, (‘NSCLC-Radiomics’, version 3: updated 23/10/2019)^28^. This dataset consists of 422 NSCLC patients treated at MAASTRO in Maastricht with curative intent radiotherapy with or without chemotherapy. One patient was excluded from analysis as the patient underwent surgery prior to radiotherapy (‘Lung1-128’). Three further patients were excluded from analysis due to data processing errors in the original MAASTRO contour file (Lung1-083, Lung1-137, Lung1-158). Radiotherapy treatment planning digital imaging and communications in medicine (DICOM) CT images and physician-delineated primary NSCLC tumours as radiotherapy structure sets were downloaded from the TCIA website and used to calculate radiomics feature values. All patients had a spiral CT scan taken of 3 mm slice thickness (voxel dimensions 1 mm × 1 mm × 3 mm). The majority of CT scans are non-contrast scans, as per a recently published study that provided a quantitative analysis which classifies scans from this dataset as contrast or non-contrast^29^. At the time of initiation of the study (October 2018 to January 2019), radiotherapy structure sets were not available for 92 patients on the TCIA website and these were manually contoured at Peter MacCallum Cancer Centre (PMCC) by a radiation oncologist (GK) with additional review by a lung radiologist (BW) as required. At the time of delineation, the radiation oncologist and radiologist did not have access to the original contours performed at MAASTRO, clinical data, or additional diagnostic imaging or reports. Delineation was performed with the original aim of replicating the work published in Aerts et al. 2014^7^. The MAASTRO contours for these 92 patients subsequently became available online in October 2019, allowing for this study to be performed.

**Figure 1:**
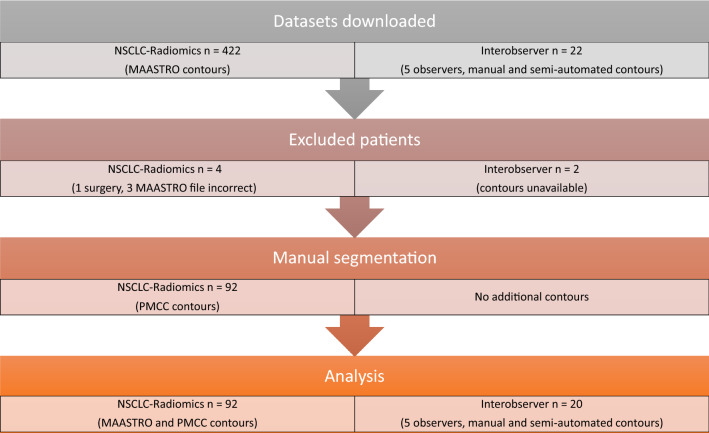
Methods flow chart. This flow chart describes the process of patient selection, creation of contours, and exclusion for the two datasets downloaded from the open access online archive ‘The Cancer Imaging Archive’: ‘NSCLC-Radiomics’ and ‘NSCLC-Radiomics-Interobserver1’ (referred to as ‘Interobserver’).

The second dataset we employed consists of 22 patients with PET confirmed non-metastatic NSCLC treated with radiotherapy (‘NSCLC-Radiomics-Interobserver1’, subsequently referred to as ‘Interobserver’)^30^. CT scans used for radiotherapy planning (spiral CT scan of the whole thorax with intravenous contrast), contours for the primary tumour and clinical data were available for 20 patients (unavailable for patient 9 and 19) and are included for radiomics analysis. For these patients, manual and semi-automated contours of the VOI were created by five radiation oncologists, resulting in a total of five manual and five semi-automated contours per patient. DICOM metadata for the CT images used for both datasets can be found in Supplementary material. Figure 1 illustrates the datasets used in this study.

### Radiomics analysis

Radiomic features were extracted utilising a free and open-source software, PyRadiomics (v3.0). Software wrapper extensions collectively known as O-RAW were used to convert DICOM objects into numerical arrays as inputs for PyRadiomics^31^. The open source PyRadiomics has standardised the feature calculation formulae^32^. Radiomic feature definitions and their compliance, or in certain cases, divergence from Image biomarker standardisation initiative (IBSI) guidelines^33^ can be found documented online (https://pyradiomics.readthedocs.io). The image pre-processing methodology involved extracting intensity in bin widths of 25 Hounsfield Units (HU), with no image resampling and no image intensity normalization. All of these settings are the default in PyRadiomics. This was performed in order to replicate the original studies using these datasets, although it is recognised this may not be consistent with current practice and recommendations by IBSI^33^. We also analysed our results using isotropic resampling to 2 × 2 × 2 mm (as per IBSI recommendations) and range re-segmentation (-300HU to + 200HU)^34^.

Radiomic features are broadly divided into three feature groups of shape (n = 16), first order (n = 19) and texture (n = 75) features. Three of these features (original_shape_Compactness1, original_shape_Compactness2 and original_firstorder_StandardDeviation) are considered redundant in PyRadiomics (v3.0), however were included in this study to allow for comparison of our results with prior studies and for completeness. First order features describe intensity histogram features of a VOI, whereas shape features describe the 3D geometric properties of the VOI. Textural features, describe spatial relationships of pixel intensities within a VOI and are further divided into symmetrical gray level co-occurrence matrix (GLCM), gray level size zone matrix (GLSZM), gray level run length matrix (GLRLM), neighbouring gray tone difference matrix (NGTDM), and gray level dependence matrix (GLDM). Additionally, wavelet and Laplacian of Gaussian (LoG) filters (sigma values 1.0, 2.0 and 3.0) were applied to the images, following which first order and texture features were subsequently recalculated, resulting in 1133 features, which were used in this analysis. Fixed bin widths of 5 and 10 were used for wavelet and LoG filters respectively. The coif1 wavelet package from the pywavelets library (v0.5.2, https://github.com/PyWavelets/pywt) was used to generate wavelet features with a starting bin edge of 0. The exact settings used for radiomics feature extraction are detailed in the parameter file (see Supplementary material).

### Statistical analysis

Statistical analysis was performed using R (v4.0.2)^35^. To study the range of IOV in tumour delineation, the Dice coefficient (DC)^36,37^ was calculated, which compares the overlap between two contours. The DC ranges between 0 and 1, with 0 indicating no spatial overlap and 1 indicating complete overlap between a pair of contours. For the ‘NSCLC-Radiomics’ dataset, there were two contours available for 92 patients (the original contour defined at MAASTRO and the second contour delineated at PMCC) with one DC calculated per patient. For the ‘Interobserver’ dataset, DC was calculated separately for the manual (n = 5) and semi-automated (n = 5) delineations, and separately for each unique pair of observers, resulting in 10 manual and 10 semi-automated DC per patient. The robustness of radiomic features to IOV was defined using the two-way (2,1) random effects intraclass correlation coefficient (ICC)^38^. ICC was computed for each radiomics feature to specify variation in results between (1) MAASTRO and PMCC contours for the ‘NSCLC-Radiomics’ dataset, and separately for the (2) five manual and (3) five semi-automated delineations within the ‘Interobserver’ dataset, resulting in three ICC values per radiomics feature. An ICC value of 0 indicates no reliability whereas a value of 1 means that the measurements are highly stable. Univariate Cox regression analysis for overall survival (OS) was performed using the radiomics features utilised in Shi et al. 2019^8^ for the subset of 92 patients from both the original MAASTRO and PMCC contours^39,40^.

## Results

### Clinical characteristics

The available clinical characteristics of patients within the ‘NSCLC-Radiomics’ and ‘Interobserver’ datasets are shown in Table 1. The ‘NSCLC-Radiomics’ dataset is further divided into patients with additional PMCC contours and patients without PMCC contours. For all three groups of patients, the median age was between 65 and 70, the majority were male, and had Stage III disease. There were some differences however in the categorisation of patients particularly across stage and histology. There were a greater proportion of patients with Stage IIIB disease in the ‘NSCLC-Radiomics’ group compared to the ‘Interobserver’ group. With respect to histology, the majority of patients with PMCC contours had squamous cell carcinoma, while patients without PMCC contours had an equal distribution of squamous and large cell carcinoma. The majority of patients in the ‘Interobserver’ group had adenocarcinoma.

**Table 1:** Clinical characteristics.

Characteristic	NSCLC-Radiomics		
	PMCC contours, N = 92	No PMCC contours, N = 329*	Interobserver, N = 22
**Age (median, IQR)**	70 (62, 75)	68 (61, 76)	67 (57, 71)
(Missing)	2	20	0
**Gender**			
Female	37 (40%)	94 (29%)	9 (41%)
Male	55 (60%)	235 (71%)	13 (59%)
**Overall stage**			
I	13 (14%)	80 (24%)	3 (14%)
II	12 (13%)	28 (8.5%)	1 (4.5%)
IIIA	35 (38%)	76 (23%)	16 (73%)
IIIB	32 (35%)	144 (44%)	2 (9.1%)
(Missing)	0	1	0
**Histology**			
Adenocarcinoma	18 (21%)	33 (11%)	10 (45%)
Large cell carcinoma	13 (15%)	101 (35%)	3 (14%)
Squamous cell carcinoma	50 (57%)	102 (35%)	6 (27%)
Other / Not specified	6 (6.9%)	56 (19%)	3 (14%)
(Missing)	5	37	0

N = number; PMCC = Peter MacCallum Cancer Centre; IQR = interquartile range; *excludes 1 patient who underwent surgery prior to radiotherapy.

### Dice coefficients

**Figure 2:**
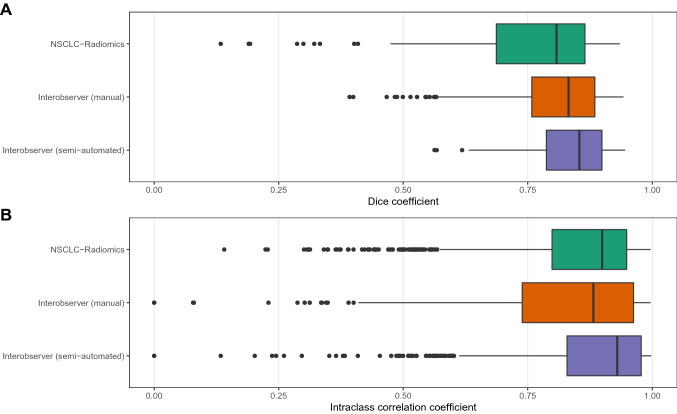
Boxplots of Dice coefficients (DC) and intraclass correlation coefficients (ICC). This figure summarises the (**A**) DC and (**B**) ICC values for the ‘NSCLC-Radiomics’, ‘Interobserver (manual)’ and ‘Interobserver (semi-automated)’ sets of contours.

For the 92 patients within the ‘NSCLC-Radiomics’ dataset that had PMCC volumes contoured, 22 volumes had a DC of less than 0.6. PMCC volumes in which the DC were less than or equal to 0.6 were reviewed. Of these, eight patients had the incorrect lesion contoured as the primary (location of primary was not available at the time of initial contour) or included the nodal volume within the VOI. These were amended to contour the correct lesion or exclude the nodal volume. The resultant DC for all datasets are shown in Fig. 2A, with the lowest median DC attributed to the ‘NSCLC-Radiomics’ dataset of 0.81 and the highest median DC of 0.85 seen in the ‘Interobserver’ (semi-automated) dataset.

### Intraclass correlation coefficient

**Figure 3:**
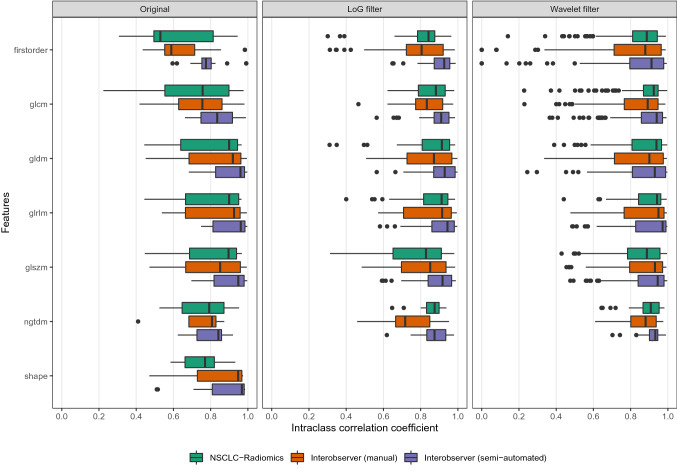
Intraclass correlation coefficients (ICC) by feature type. This figure separately considers the ICC values of radiomics features by different classes of features, as well as with the addition of Laplacian of Gaussian (LoG) and wavelet filters for the ‘NSCLC-Radiomics’, ‘Interobserver (manual)’ and ‘Interobserver (semi-automated)’ sets of contours.

The median two-way random effects ICC values for all radiomics features for the ‘NSCLC-Radiomics’, ‘Interobserver’ (manual) and ‘Interobserver’ (semi-automated) contours were 0.90, 0.88 and 0.93 (Fig. 2B). The ICC varied according to the feature type (see Fig. 3). For all datasets the ICC was highest for the texture features GLDM, GLRLM, GLSZM, and NGTDM, and generally lower for first order and GLCM features. Shape features had a lower median ICC in the ‘NSCLC-Radiomics’ dataset compared to the ‘Interobserver’ dataset. For all original feature types, ‘Interobserver’ (semi-automated) contours had the highest median ICC. The addition of LoG and wavelet filters (applied to all apart from shape features) increased the median ICC value of most feature types, although within some groups a few outlier features with lower ICCs are seen. Excluding patients with DC of less than 0.6 from the ‘NSCLC-Radiomics’ dataset increased the median ICC of feature types, in particular shape features, however first order and GLCM still had lower median ICC values compared to the other feature types (Supplementary material, Fig. 1). With respect to the outliers, over 200 radiomics features had an ICC of less than 0.6 in any of the datasets, with 64 of these features appearing in multiple datasets (Supplementary material, Tables 1, 2 and Fig. 2). Interestingly the feature ‘original_shape_Compactness2’, which is a feature that is represented in the Aerts et al. 2014 radiomics signature^7^ has a low ICC for both the ‘Interobserver’ (manual) and ‘Interobserver’ (semi-automated) contours (0.47 and 0.51 respectively). The radiomic features (with or without a filter) with an ICC of less than 0.6 to most commonly be present in any of the datasets were ‘firstorder_mean’ (n = 14), ‘firstorder_Skewness’ (n = 13), ‘firstorder_RootMeanSquared’ (n = 11), ‘glcm_MCC (maximum correlation coefficient)’ (n = 13), and ‘gldm_LargeDependenceLowGrayLevelEmphasis’ (n = 10) (Supplementary material, Table 3). ICC results by feature type and the number and type of features with a low ICC of < 0.6 within the ‘NSCLC-Radiomics’ dataset did not differ after resampling and re-segmentation of radiomics features as per IBSI guidelines (Supplementary material, Fig. 3 and Table 4).

### Relationship between dice coefficients and intraclass correlation coefficients

**Figure 4:**
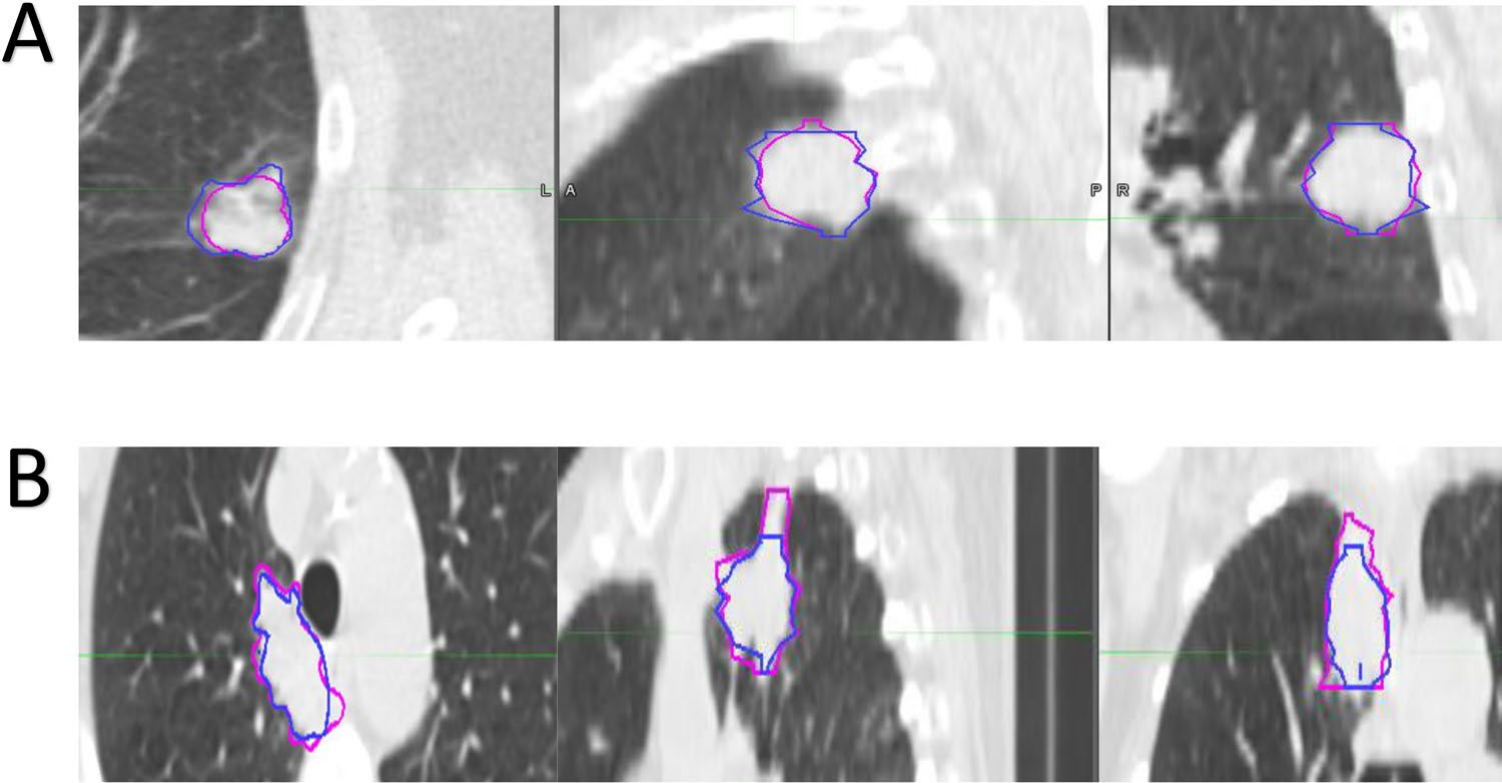
Case examples. Two examples (patient A and B) from the ‘NSCLC-Radiomics’ dataset are provided highlighting patients who had high Dice coefficients (DC) yet also had relatively high absolute differences in the shape feature ‘original_shape_Compactness2’ (A = Lung1-157, DC = 0.90, Difference = 0.15; B = Lung1-378, DC = 0.79, Difference = 0.12). Note blue = MAASTRO contour and pink = PMCC contour.

A previously published prognostic signature developed using the ‘NSCLC-Radiomics (n = 421)’ dataset found four radiomics features to be prognostic for overall survival: original_firstorder_Energy, original_glrlm_GrayLevelNonUniformity, wavelet-HLH_glrlm_GrayLevelNonUniformity and original_shape_Sphericity^3 (named ‘original_shape_Compactness2’ in PyRadiomics v3.0)^8^. All features have a very high ICC (0.91, 0.97 and 0.95 respectively) apart from ‘original_shape_Compactness2’ which has a lower ICC of 0.61 within the ‘NSCLC-Radiomics’ dataset. Bland–Altman plots for these four features generally show that tumour volumes with high DC had low absolute differences in the value of the radiomics feature, while lower DC had higher absolute differences (see Supplementary material, Fig. 4). Examples of outliers, in which tumour contours had high DC, yet also had a relatively high absolute difference in the result of the radiomics feature ‘original_shape_Compactness2’ are shown (Fig. 4).

### Survival analysis

**Figure 5:**
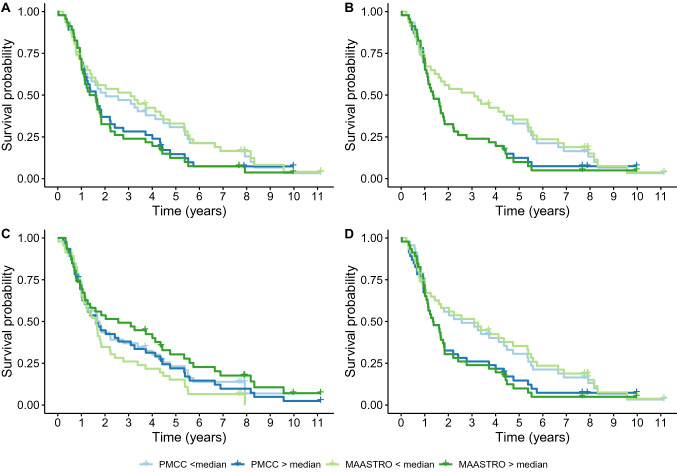
Kaplan Meier survival curves. Overall survival curves for MAASTRO and PMCC contours from the ‘NSCLC-Radiomics’ dataset are given for four radiomics features found to be prognostic in a previously published model, showing similar separation of curves for three features (A, B and D), while feature C shows separation of curves for the MAASTRO contour, however not for the PMCC contour (A = original_firstorder_Energy, B = original_glrlm_GrayLevelNonUniformity, C = original_shape_Compactness2, D = wavelet-HLH_glrlm_GrayLevelNonUniformity).

Survival analysis was performed as per the original Aerts et al. 2014 analysis^7^. The median value of the four radiomics features was calculated separately for the original MAASTRO and PMCC contours on the ‘NSCLC-Radiomics’ dataset (see Supplementary material, Table 5). Univariate Kaplan–Meier survival curves and hazard ratios for both sets of contours were similar for all features apart from ‘original_shape_Compactness2’, which had the lowest ICC value of 0.61 amongst the four features, and for which the survival curves did not separate when using the PMCC contours (see Fig. 5 and Supplementary material, Fig. 5 and Table 6). For this feature, the hazard ratio for survival was significant for the MAASTRO contours (HR 0.79, 95% CI 0.63–0.99, *p*-value = 0.04), however the hazard ratio is notably higher and loses statistical significance for survival for the PMCC contours (HR 0.90, 95% CI 0.72–1.12, *p*-value = 0.34). The feature with the highest ICC, original_glrlm_GrayLevelNonUniformity, has a statistically significant hazard ratio for survival on the MAASTRO contours (HR 1.31, 95% CI 1.03–1.67, *p*-value = 0.03) and maintains this using the PMCC contours (HR 1.28, 95% CI 1.00–1.63, *p*-value = 0.05). The above findings suggest that features that are highly robust to IOV in delineation are less likely to lose their prognostic ability, while features that are less robust, are more likely to lose their prognostic ability with contour variation.

## Discussion

This study has shown that the majority of radiomics features are robust to IOV in contouring in the setting of NSCLC being treated with radical intent radiotherapy, with a high median ICC for radiomics features found in both the ‘NSCLC-Radiomics’ and ‘Interobserver’ datasets. In particular, texture features GLDM, GLRLM, GLSZM, and NGTDM were the most robust, while first order and GLCM features were the least robust to IOV in delineation. The robustness of shape features varied according to the dataset, with a lower median ICC in the ‘NSCLC-Radiomics’ dataset compared to the ‘Interobserver’ dataset. For all original feature types, contours generated using semi-automated methods were the most robust. The addition filters generally increased the robustness of most feature types. To our knowledge, this is the first study to also suggest for features that are less robust to IOV in contouring, defined by their lower ICC values, variation in contouring may affect the features’ prognostic ability and therefore should be used with caution in prognostic models predicting survival. The results of this study may also at least partly provide an explanation for the lack of reproducibility of radiomics based prognostic models, showing that variation in contouring alone, may lead to differences in some radiomics features that are significant enough to result in these features no longer being able to predict survival. In this study, we therefore also provide a detailed list of radiomics features that are not robust to IOV in delineation, which may help guide future studies in selecting and excluding features to be used within prognostic models.

Textural features were the group most robust to variations in contouring, while first order and shape features were more sensitive to changes in contour. As suggested by the examples in Fig. 4, the inclusion of regions surrounding the tumour on CT scan which are often highly subjective, such as vessels, spiculation or ground glass changes, or areas of pleural attachment or surrounding atelectasis, may significantly change the overall smoothness of the contour, and resultant shape of the contour. Additionally, decisions to use certain features on contouring software, such as interpolation between slices which may result in a smoother contour versus contouring slice by slice, may also impact upon the radiomics result of the shape feature. These results are similar to the findings of a study by Pavic et al. 2018 which assessed IOV in contouring in patients with lung cancer, mesothelioma and head and neck squamous cell carcinoma (HNSCC) and found an overall high stability rate of radiomics features (defined as ICC > 0.8) in lung cancer (90% of radiomics features), with shape features having the lowest ICC in all tumour sites^22^. Interestingly the stability rate was considerably lower for HNSCC (59%) and mesothelioma (36%) suggesting robustness of radiomics features to IOV in contouring is tumour site dependent. Another study in 20 patients with lung cancer found that manual segmentation resulted in the lowest ICC for first order radiomics features (ICC = 0.63) compared to textural (ICC = 0.82) and shape (ICC = 0.80) features^19^. In contrast, however, one lung cancer study did not find any particular class of radiomics features to be less robust to contouring variation^21^, and another study in patients with lung, kidney and liver malignancies, found shape and first order features to be the most robust to variation in contours^24^. The difference in results of the latter study compared to our findings may partly reflect the different patient population and tumour sites in this study, as well as the use of a ‘lung nodules’ imaging dataset, that captured all lesions within the lung, some of which may not have been malignant tumours. Ultimately, the robustness of a feature to IOV should be taken into account when developing radiomics based prognostic models, as contouring variation for less robust features may affect their prognostic performance, as seen with feature ‘original_shape_Compactness2’, which appeared to lose its prognostic power using PMCC contours.

This study also showed that improvement in DC usually resulted in greater agreement in radiomics feature results. Measures to reduce contouring variation therefore should be considered in radiomics studies to improve reproducibility of results. One method that may reduce contouring variation is the use of semi-automated techniques such as that used within the ‘Interobserver’ dataset, which resulted in both an improvement in DC and ICC of radiomics features. This technique has also been shown to improve contour and radiomics results agreement in other studies^10,19,21^. Another potential way to decrease IOV includes ensuring availability of all clinical and diagnostic information to clinicians creating contours. The consistency of shape features, in particular, may be improved through ensuring data such as biopsy, staging, diagnostic CT scans and PET images are available at the time of contouring. While this is usually the case, this may become more challenging when using open access online repositories, as used in this study, or when imaging data is shared across departments and institutions for the purpose of creating contours to be used in radiomics research. Certainly, in this study, DC were higher in the ‘Interobserver’ versus ‘NSCLC-Radiomics’ dataset, in which the observers using the former dataset had access to the same set of information during delineation of contours, compared to no additional information available upon delineation of the PMCC contours within the ‘NSCLC-Radiomics’ dataset. This finding is further supported by a systematic review that investigated the repeatability and reproducibility of radiomics features and found that shape metrics using PET images appeared to be less subject to IOV^10^. Finally, the use of contouring guidelines, where available, should be utilised and may also reduce contouring variation. Various solutions may also exist to mitigate the impact of IOV in contouring on radiomics feature results following completion of contours. Use of filters, as shown in this study, may improve the ICC of radiomics features and reduce the impact of contour variation. Post-processing of contours, including exclusion of voxels with very high or low HU on CT that may clearly indicate bone or air, may improve the accuracy of contours, although did not improve the robustness of features within this study.

While the improvement in consistency across contours may improve robustness of radiomics features, it also raises the challenging question of what constitutes the ‘ground truth’ with respect to the radiological tumour volume. Interrogation of imaging of surgical patients and correlation with histopathological findings may be helpful, however not a feasible approach in non-surgical cases, and has inherent limitations due to the processing methods required of tissue prior to histopathological analysis. Contouring guidelines may improve consistency across observers, however, these guidelines are largely developed for the purposes of radiotherapy treatment and may not be entirely ‘fit for purpose’ for radiomics research. Radiotherapy treatment contours are generally developed in order to minimise the risk of tumour miss, and in some cases may include tumour motion, resulting in larger contours, whereas for radiomics research, it may be more important to exclude areas in which there is uncertainty regarding tumour involvement. The choice of approach may result in affect the radiomics features of the tumour, including shape features. Furthermore, it is yet to be determined whether the entire tumour, or certain regions of the tumour have radiomics features relevant for particular endpoints. There is increasing evidence that the peri-tumoural zone may be significant in determining prognostic endpoints such as locoregional recurrence, response to treatment and survival^41–45^. Apart from dividing the tumour into regional zones, it may be beneficial to recognise the inherent uncertainties in creating a VOI, and to conceptualise ‘probabilistic’ contours, either through in silico modelling or through ‘expert’ input, to take into account the estimated odds that a certain region represents tumour, and to subsequently interrogate the radiomics features of these different regions. This could also be performed by considering the ‘overlap’ region of multiple contours of a tumour as a representation of the ‘ground truth’, although this would be a labour intensive approach. Ultimately, the elimination of the need for a human derived contour, through approximation of a volume of interest by use of a seed point within the tumour and creation of a 3D square area indicating a region of interest may also be utilised in conjunction with deep learning techniques. This is a promising approach that is currently being utilised and investigated in the literature^46–49^. The validity of all these approaches, however, require further exploration in a systematic way.

Limitations of this study include the use of a single observer for creation of PMCC contours and lack of availability of all diagnostic imaging and pathology reports at our institution. Whilst this limitation may have reduced consistency between contours for a given patient, the resultant greater range in DC allowed us to more fully explore the impact of IOV in contouring on radiomics features, as well as postulate regarding differences seen between the ‘NSCLC-Radiomics’ and ‘Interobserver’ datasets given the differences in methodology. Any effect of intra-observer variation in delineation has not been studied. The findings from this study may also not be applicable to different tumour histologies and stages, and to different imaging modalities, including contours developed on imaging other than radiotherapy planning scans and by craft groups other than radiation oncologists, however the homogenous nature of our patient dataset, strengthens the applicability of our study findings in patients with lung cancer. Our sample size was relatively small for the purpose of determining statistically significant associations of radiomics features with survival, and for this reason, the four features found to be prognostic in the Aerts et al. 2014 study^7^, were chosen to compare results of survival analysis between MAASTRO and PMCC contours. It is recognised however that some of these features have underlying volume dependencies and may not provide significant additional prognostic information over volume alone^50^. Nevertheless, the primary objective of this study was to assess the robustness of radiomics features to IOV in contouring, rather than to generate a new prognostic signature. Moreover, we were able to highlight the significance of selecting robust radiomics features within survival models, which retain their prognostic ability despite contours being created by different clinicians. Additionally, to our knowledge, this is the largest IOV study within the radiomics literature in patients with histologically proven lung cancer.

## Conclusions

In conclusion, we found the vast majority of radiomics features were robust to IOV in delineation of primary NSCLC treated with radiotherapy. Overall, first order and GLCM were the least robust, with the lowest median ICC across all datasets. These results were overall improved with the addition of LoG and wavelet filters. Three of the four radiomics features used within a prognostic signature for NSCLC patients had very high ICC of greater than 0.90. The shape feature ‘Compactness2’ was less robust to IOV in contouring and caution may be applied to its use. The results of this study may further guide appropriate selection of radiomics features within prognostic models in patients with NSCLC. Additionally, this study supports actions to reduce IOV within radiomics studies including ensuring availability of all clinical and diagnostic imaging at the time of contouring, and consideration given to use of semi-automated techniques.

## Supplementary Information

Supplementary Information 1.

Supplementary Information 2.

Supplementary Information 3.

Supplementary Information 4.

Supplementary Information 5.

Supplementary Information 6.

Supplementary Information 7.

Supplementary Information 8.

Supplementary Information 9.

Supplementary Information 10.

Supplementary Information 11.

Supplementary Information 12.

Supplementary Information 13.

Supplementary Information 14.

Supplementary Information 15.

## Data Availability

The de-identified imaging and clinical datasets analysed during the current study are available from an openly accessible online centralised repository, The Cancer Imaging Archive (TCIA) (https://www.cancerimagingarchive.net)^4,6^. Additional data used and analysed during the current study are available from the corresponding author on reasonable request.

## Abbreviations

DC: Dice coefficients
DICOM: Digital imaging and communications in medicine
GLCM: Gray level co-occurrence matrix
GLDM: Gray level dependence matrix
GLRLM: Gray level run length matrix
GLSZM: Gray level size zone matrix
HNSCC: Head and neck squamous cell carcinoma
HU: Hounsfield units
IBSI: Image biomarker standardisation initiative
ICC: Intraclass correlation coefficient
IOV: Inter-observer variation
LoG: Laplacian of Gaussian
NGTDM: Neighbouring gray tone difference matrix
NSCLC: Non-small cell lung cancer
OS: Overall survival
PMCC: Peter MacCallum Cancer Centre
RF: Radiomics features
TCIA: The cancer imaging archive
VOI: Volume of interest
