# Supplementary figures and images for: The impact of inter-observer variation in delineation on robustness of radiomics features in non-small cell lung cancer
Source: Sci Rep. 2022 Jul 27;12:12822. doi: 10.1038/s41598-022-16520-9 (PMC9329346; doi:10.1038/s41598-022-16520-9)

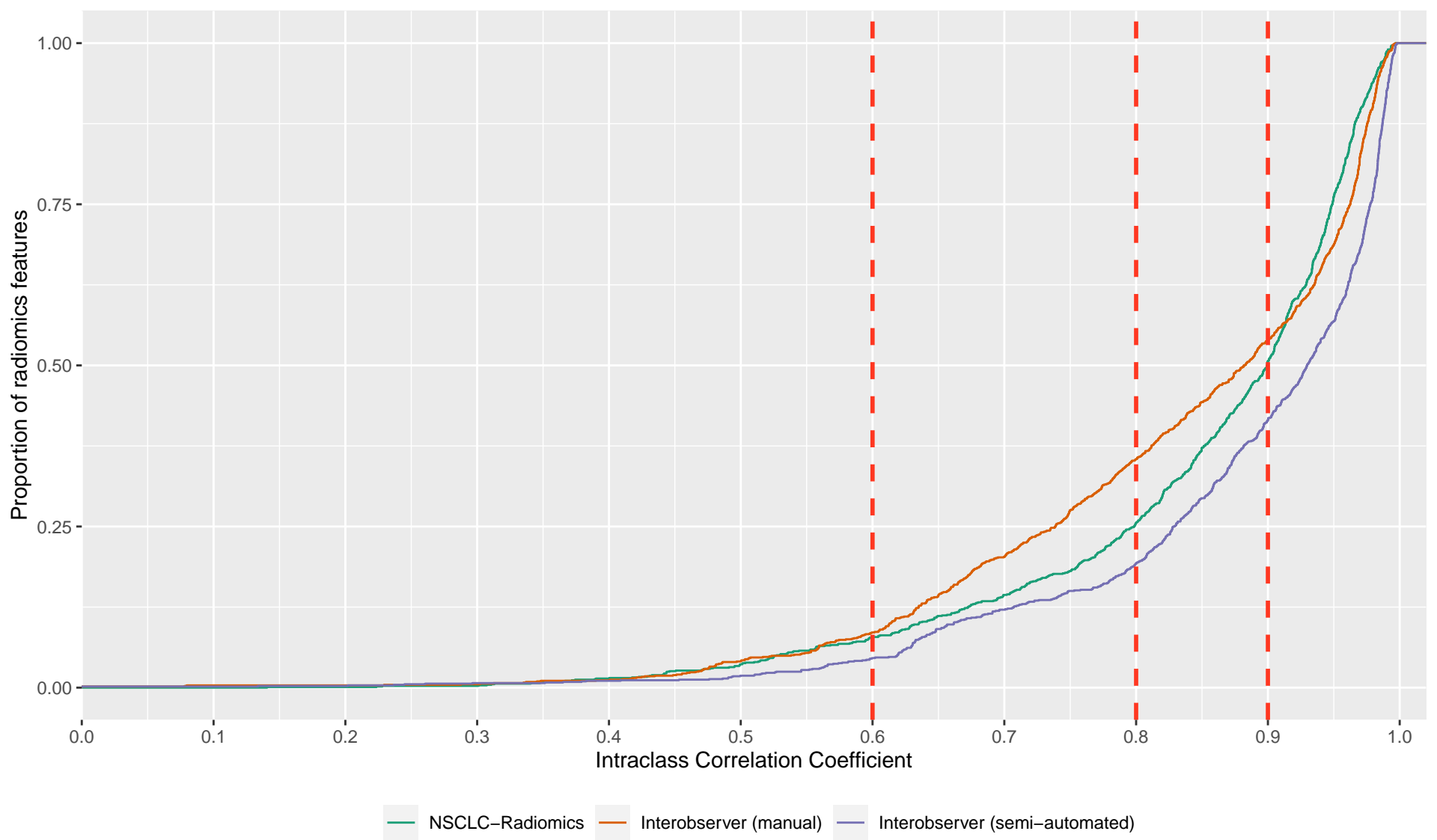

Supplement: Supplementary file 1 — Supplementary Information 1. [file 41598_2022_16520_MOESM1_ESM.pdf]

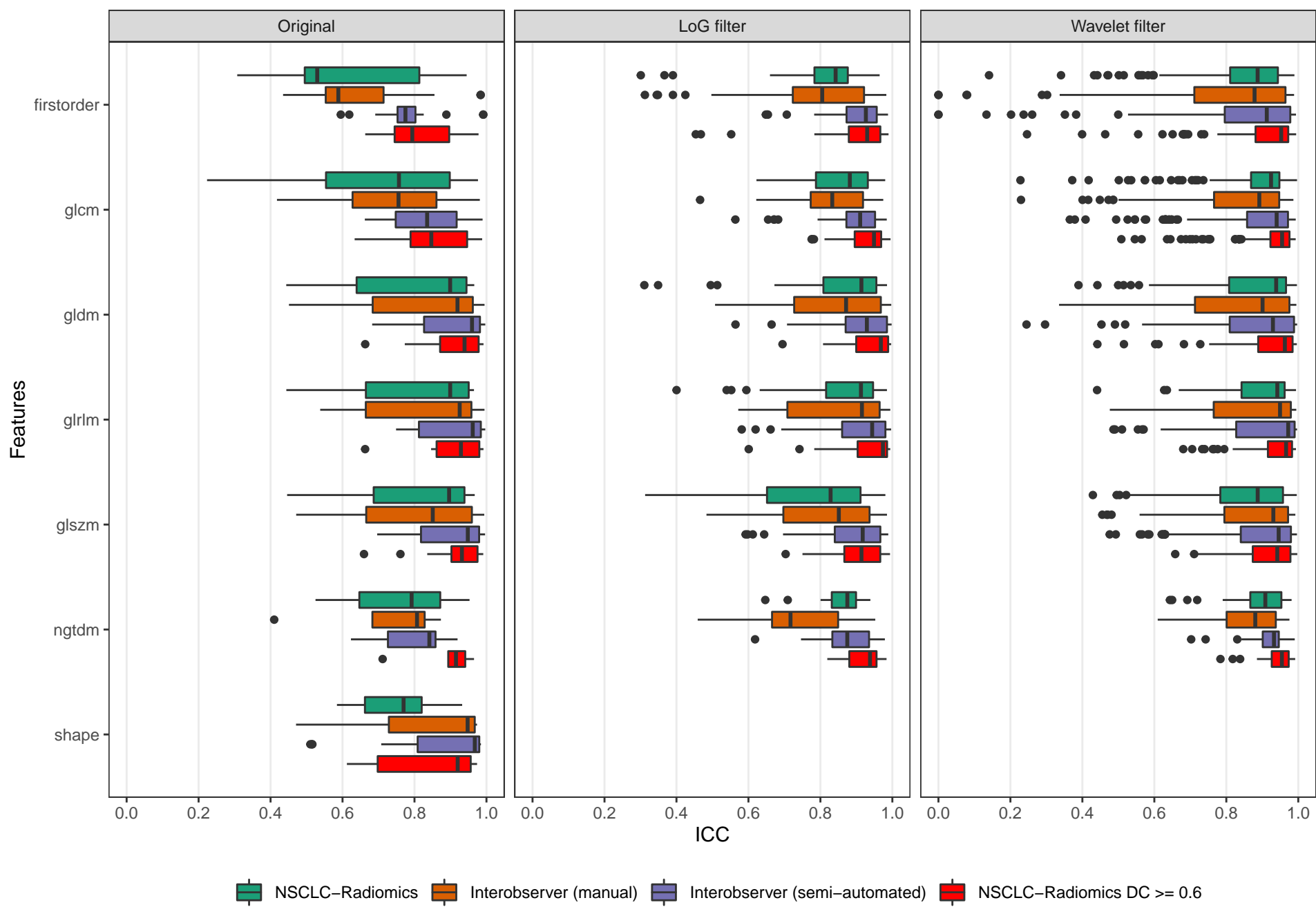

Supplement: Supplementary file 5 — Supplementary Information 5. [file 41598_2022_16520_MOESM5_ESM.pdf]

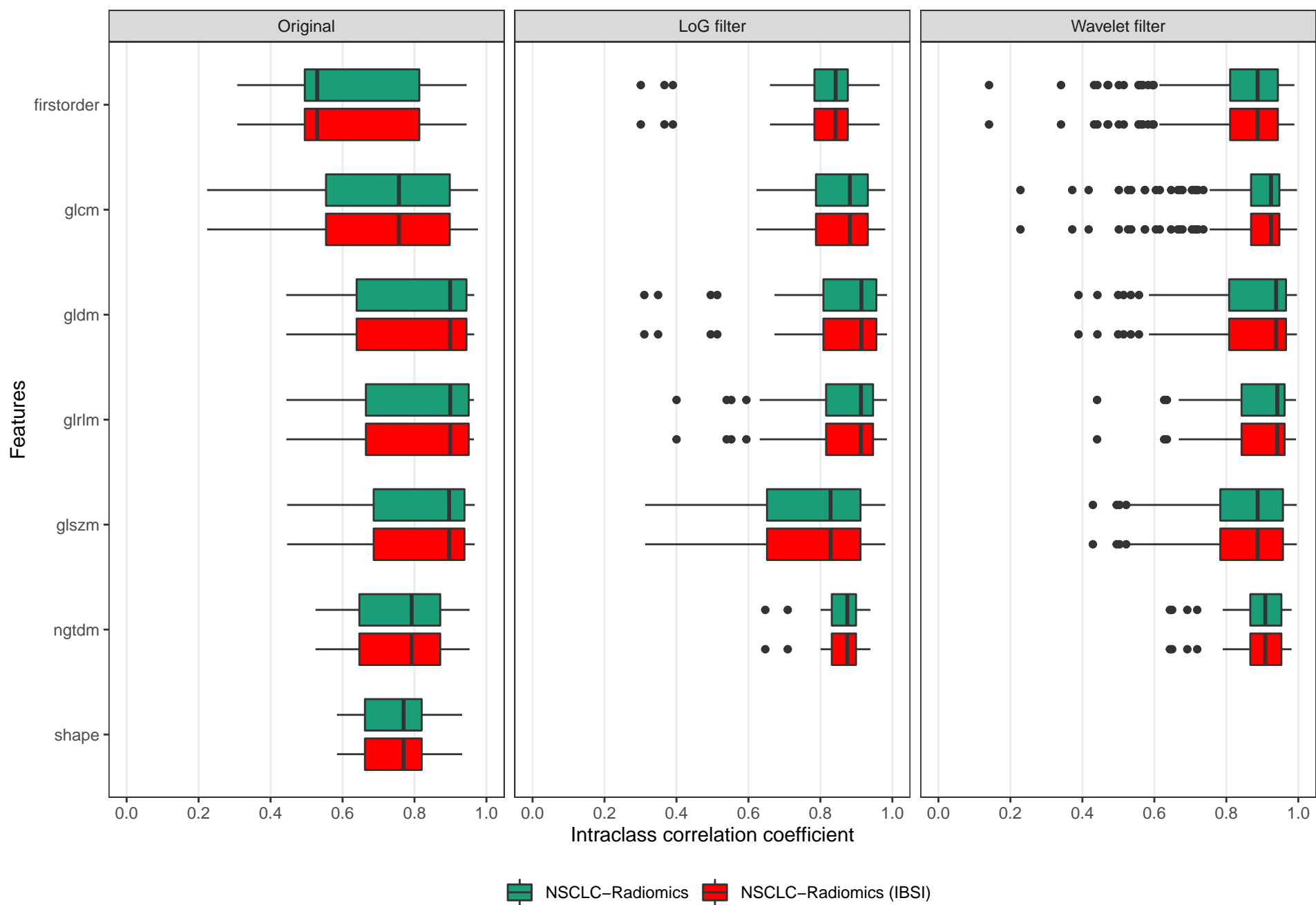

Supplement: Supplementary file 6 — Supplementary Information 6. [file 41598_2022_16520_MOESM6_ESM.pdf]
