# Supplementary material for: The impact of inter-observer variation in delineation on robustness of radiomics features in non-small cell lung cancer
Source: Sci Rep. 2022 Jul 27;12:12822. doi: 10.1038/s41598-022-16520-9 (PMC9329346; doi:10.1038/s41598-022-16520-9)
Supplement: Supplementary file 2 — Supplementary Information 2. [file 41598_2022_16520_MOESM2_ESM.docx]

**Supplementary Table 1: Features with intraclass correlation coefficient of less than 0.6**

| **Feature** | **Dataset** | **ICC** |
| --- | --- | --- |
| wavelet-HHH_firstorder_Mean | Interobserver (manual) | 0.000 |
| wavelet-HHH_firstorder_Mean | Interobserver (semi-automated) | 0.000 |
| wavelet-LHH_firstorder_Mean | Interobserver (manual) | 0.000 |
| wavelet-LHH_firstorder_Mean | Interobserver (semi-automated) | 0.000 |
| wavelet-HHH_firstorder_RootMeanSquared | Interobserver (manual) | 0.078 |
| wavelet-LHH_firstorder_RootMeanSquared | Interobserver (manual) | 0.079 |
| wavelet-HLH_firstorder_RootMeanSquared | Interobserver (semi-automated) | 0.133 |
| wavelet-HHH_firstorder_Mean | NSCLC-Radiomics | 0.140 |
| wavelet-HHH_firstorder_Skewness | Interobserver (semi-automated) | 0.201 |
| original_glcm_ClusterShade | NSCLC-Radiomics | 0.223 |
| wavelet-LLL_glcm_ClusterShade | NSCLC-Radiomics | 0.227 |
| wavelet-LLH_glcm_Correlation | Interobserver (manual) | 0.229 |
| wavelet-HHH_firstorder_RootMeanSquared | Interobserver (semi-automated) | 0.236 |
| wavelet-LLH_gldm_LargeDependenceLowGrayLevelEmphasis | Interobserver (semi-automated) | 0.244 |
| wavelet-LHH_firstorder_RootMeanSquared | Interobserver (semi-automated) | 0.260 |
| wavelet-HLH_firstorder_RootMeanSquared | Interobserver (manual) | 0.287 |
| wavelet-LLL_gldm_LargeDependenceLowGrayLevelEmphasis | Interobserver (semi-automated) | 0.296 |
| log-sigma-2-0-mm-3D_firstorder_90Percentile | NSCLC-Radiomics | 0.300 |
| wavelet-HLH_firstorder_Mean | Interobserver (manual) | 0.302 |
| original_firstorder_Median | NSCLC-Radiomics | 0.307 |
| log-sigma-2-0-mm-3D_gldm_DependenceNonUniformity | NSCLC-Radiomics | 0.310 |
| log-sigma-2-0-mm-3D_firstorder_90Percentile | Interobserver (manual) | 0.312 |
| log-sigma-2-0-mm-3D_glszm_SizeZoneNonUniformity | NSCLC-Radiomics | 0.312 |
| wavelet-LLL_gldm_LargeDependenceLowGrayLevelEmphasis | Interobserver (manual) | 0.335 |
| wavelet-HHH_firstorder_Skewness | Interobserver (manual) | 0.336 |
| wavelet-HHH_firstorder_RootMeanSquared | NSCLC-Radiomics | 0.340 |
| log-sigma-3-0-mm-3D_firstorder_Kurtosis | Interobserver (manual) | 0.345 |
| log-sigma-1-0-mm-3D_glszm_SizeZoneNonUniformity | NSCLC-Radiomics | 0.347 |
| log-sigma-3-0-mm-3D_firstorder_Skewness | Interobserver (manual) | 0.348 |
| log-sigma-3-0-mm-3D_gldm_DependenceNonUniformity | NSCLC-Radiomics | 0.349 |
| wavelet-LHH_firstorder_Skewness | Interobserver (semi-automated) | 0.351 |
| log-sigma-3-0-mm-3D_glszm_SizeZoneNonUniformity | NSCLC-Radiomics | 0.364 |
| wavelet-HHH_glcm_ClusterShade | Interobserver (semi-automated) | 0.365 |
| log-sigma-1-0-mm-3D_firstorder_90Percentile | NSCLC-Radiomics | 0.366 |
| wavelet-LLL_glcm_ClusterTendency | NSCLC-Radiomics | 0.371 |
| original_glcm_ClusterTendency | NSCLC-Radiomics | 0.374 |
| wavelet-HHL_glcm_ClusterShade | Interobserver (semi-automated) | 0.379 |
| wavelet-HLH_firstorder_Mean | Interobserver (semi-automated) | 0.382 |
| wavelet-LHL_gldm_LargeDependenceLowGrayLevelEmphasis | NSCLC-Radiomics | 0.389 |
| log-sigma-3-0-mm-3D_firstorder_90Percentile | NSCLC-Radiomics | 0.390 |
| log-sigma-1-0-mm-3D_firstorder_90Percentile | Interobserver (manual) | 0.390 |
| log-sigma-3-0-mm-3D_glrlm_RunEntropy | NSCLC-Radiomics | 0.400 |
| wavelet-HHH_glcm_ClusterShade | Interobserver (manual) | 0.400 |
| wavelet-LLH_glcm_Correlation | Interobserver (semi-automated) | 0.408 |
| original_ngtdm_Complexity | Interobserver (manual) | 0.410 |
| wavelet-LLL_glcm_MaximumProbability | Interobserver (manual) | 0.416 |
| wavelet-LLL_glcm_SumSquares | NSCLC-Radiomics | 0.417 |
| original_glcm_MCC | Interobserver (manual) | 0.417 |
| wavelet-LHL_gldm_SmallDependenceHighGrayLevelEmphasis | Interobserver (manual) | 0.421 |
| original_glcm_SumSquares | NSCLC-Radiomics | 0.422 |
| log-sigma-3-0-mm-3D_firstorder_90Percentile | Interobserver (manual) | 0.424 |
| wavelet-HLH_firstorder_Skewness | Interobserver (manual) | 0.427 |
| wavelet-LLL_glszm_GrayLevelVariance | NSCLC-Radiomics | 0.429 |
| original_firstorder_Minimum | NSCLC-Radiomics | 0.430 |
| wavelet-LLL_firstorder_Median | NSCLC-Radiomics | 0.432 |
| original_firstorder_Skewness | Interobserver (manual) | 0.434 |
| wavelet-LLL_glrlm_GrayLevelVariance | NSCLC-Radiomics | 0.440 |
| wavelet-LLL_firstorder_Variance | NSCLC-Radiomics | 0.441 |
| wavelet-LLL_gldm_GrayLevelVariance | NSCLC-Radiomics | 0.441 |
| original_gldm_GrayLevelVariance | NSCLC-Radiomics | 0.443 |
| original_firstorder_Variance | NSCLC-Radiomics | 0.443 |
| original_glrlm_GrayLevelVariance | NSCLC-Radiomics | 0.443 |
| original_glszm_GrayLevelVariance | NSCLC-Radiomics | 0.445 |
| wavelet-HLL_glcm_MCC | Interobserver (manual) | 0.448 |
| original_firstorder_Mean | NSCLC-Radiomics | 0.450 |
| original_gldm_DependenceEntropy | Interobserver (manual) | 0.451 |
| wavelet-LHL_gldm_SmallDependenceHighGrayLevelEmphasis | Interobserver (semi-automated) | 0.453 |
| wavelet-LHL_glszm_SmallAreaHighGrayLevelEmphasis | Interobserver (manual) | 0.455 |
| original_firstorder_Minimum | Interobserver (manual) | 0.455 |
| log-sigma-1-0-mm-3D_ngtdm_Complexity | Interobserver (manual) | 0.459 |
| original_glcm_SumEntropy | Interobserver (manual) | 0.461 |
| log-sigma-1-0-mm-3D_glcm_MCC | Interobserver (manual) | 0.465 |
| wavelet-HHH_glszm_SmallAreaEmphasis | Interobserver (manual) | 0.468 |
| wavelet-HHH_firstorder_Skewness | NSCLC-Radiomics | 0.470 |
| wavelet-HHH_glszm_SizeZoneNonUniformityNormalized | Interobserver (manual) | 0.470 |
| original_shape_Compactness2 | Interobserver (manual) | 0.470 |
| original_glszm_ZoneEntropy | Interobserver (manual) | 0.471 |
| log-sigma-2-0-mm-3D_glszm_GrayLevelNonUniformity | NSCLC-Radiomics | 0.471 |
| wavelet-LLL_firstorder_Minimum | NSCLC-Radiomics | 0.471 |
| wavelet-HHL_glcm_ClusterShade | Interobserver (manual) | 0.472 |
| original_shape_Compactness1 | Interobserver (manual) | 0.475 |
| wavelet-LHL_glszm_SmallAreaHighGrayLevelEmphasis | Interobserver (semi-automated) | 0.475 |
| wavelet-LLH_firstorder_Skewness | Interobserver (manual) | 0.476 |
| wavelet-LHL_glrlm_ShortRunHighGrayLevelEmphasis | Interobserver (manual) | 0.476 |
| original_shape_Sphericity | Interobserver (manual) | 0.476 |
| log-sigma-2-0-mm-3D_glszm_GrayLevelVariance | NSCLC-Radiomics | 0.476 |
| log-sigma-2-0-mm-3D_glszm_GrayLevelNonUniformityNormalized | NSCLC-Radiomics | 0.478 |
| wavelet-LLH_gldm_LargeDependenceLowGrayLevelEmphasis | Interobserver (manual) | 0.480 |
| wavelet-LHL_glszm_HighGrayLevelZoneEmphasis | Interobserver (manual) | 0.481 |
| wavelet-LHL_glrlm_HighGrayLevelRunEmphasis | Interobserver (manual) | 0.481 |
| wavelet-LHL_gldm_HighGrayLevelEmphasis | Interobserver (manual) | 0.481 |
| log-sigma-2-0-mm-3D_glszm_GrayLevelVariance | Interobserver (manual) | 0.483 |
| wavelet-LLL_glcm_MCC | Interobserver (manual) | 0.485 |
| wavelet-LHL_glcm_Autocorrelation | Interobserver (manual) | 0.486 |
| log-sigma-1-0-mm-3D_glszm_SmallAreaLowGrayLevelEmphasis | Interobserver (manual) | 0.486 |
| wavelet-LHL_glrlm_ShortRunHighGrayLevelEmphasis | Interobserver (semi-automated) | 0.486 |
| wavelet-LHL_gldm_HighGrayLevelEmphasis | Interobserver (semi-automated) | 0.490 |
| wavelet-LHL_glrlm_HighGrayLevelRunEmphasis | Interobserver (semi-automated) | 0.490 |
| original_firstorder_90Percentile | NSCLC-Radiomics | 0.491 |
| wavelet-LHL_glszm_HighGrayLevelZoneEmphasis | Interobserver (semi-automated) | 0.492 |
| wavelet-LHL_glcm_Autocorrelation | Interobserver (semi-automated) | 0.493 |
| log-sigma-1-0-mm-3D_gldm_DependenceNonUniformity | NSCLC-Radiomics | 0.495 |
| wavelet-LLL_glszm_ZoneEntropy | NSCLC-Radiomics | 0.495 |
| log-sigma-2-0-mm-3D_firstorder_Skewness | Interobserver (manual) | 0.497 |
| original_firstorder_RootMeanSquared | NSCLC-Radiomics | 0.498 |
| original_firstorder_InterquartileRange | NSCLC-Radiomics | 0.499 |
| wavelet-LHH_gldm_LargeDependenceLowGrayLevelEmphasis | NSCLC-Radiomics | 0.499 |
| wavelet-LHH_firstorder_Kurtosis | Interobserver (semi-automated) | 0.499 |
| wavelet-HHL_firstorder_Mean | Interobserver (manual) | 0.500 |
| wavelet-HLH_glcm_ClusterShade | Interobserver (manual) | 0.500 |
| wavelet-LLL_firstorder_Mean | NSCLC-Radiomics | 0.501 |
| wavelet-LLL_glcm_MCC | NSCLC-Radiomics | 0.501 |
| wavelet-LHH_firstorder_Skewness | Interobserver (manual) | 0.502 |
| wavelet-LHL_glrlm_LongRunHighGrayLevelEmphasis | Interobserver (manual) | 0.503 |
| wavelet-LLL_glszm_SizeZoneNonUniformity | NSCLC-Radiomics | 0.503 |
| wavelet-LLL_glcm_JointEnergy | Interobserver (manual) | 0.505 |
| wavelet-HLH_gldm_LargeDependenceLowGrayLevelEmphasis | Interobserver (manual) | 0.506 |
| log-sigma-1-0-mm-3D_gldm_SmallDependenceHighGrayLevelEmphasis | Interobserver (manual) | 0.507 |
| wavelet-LHL_glrlm_LongRunHighGrayLevelEmphasis | Interobserver (semi-automated) | 0.510 |
| original_firstorder_RobustMeanAbsoluteDeviation | NSCLC-Radiomics | 0.510 |
| original_shape_Compactness2 | Interobserver (semi-automated) | 0.510 |
| log-sigma-1-0-mm-3D_gldm_LargeDependenceLowGrayLevelEmphasis | NSCLC-Radiomics | 0.513 |
| wavelet-LLL_gldm_DependenceNonUniformity | NSCLC-Radiomics | 0.514 |
| original_shape_Compactness1 | Interobserver (semi-automated) | 0.514 |
| wavelet-LLL_firstorder_90Percentile | NSCLC-Radiomics | 0.514 |
| original_shape_Sphericity | Interobserver (semi-automated) | 0.516 |
| wavelet-LHL_gldm_LargeDependenceLowGrayLevelEmphasis | Interobserver (manual) | 0.516 |
| log-sigma-3-0-mm-3D_glszm_GrayLevelNonUniformityNormalized | NSCLC-Radiomics | 0.518 |
| wavelet-LHL_gldm_LargeDependenceLowGrayLevelEmphasis | Interobserver (semi-automated) | 0.519 |
| original_glcm_JointAverage | NSCLC-Radiomics | 0.520 |
| original_glcm_SumAverage | NSCLC-Radiomics | 0.520 |
| wavelet-HLH_glcm_MCC | Interobserver (manual) | 0.521 |
| wavelet-HLL_glszm_SizeZoneNonUniformity | NSCLC-Radiomics | 0.521 |
| wavelet-HLH_firstorder_Median | Interobserver (manual) | 0.523 |
| original_firstorder_StandardDeviation | NSCLC-Radiomics | 0.524 |
| original_ngtdm_Complexity | NSCLC-Radiomics | 0.524 |
| log-sigma-3-0-mm-3D_glszm_GrayLevelNonUniformity | NSCLC-Radiomics | 0.525 |
| log-sigma-3-0-mm-3D_glszm_GrayLevelVariance | NSCLC-Radiomics | 0.525 |
| wavelet-LLL_glcm_MCC | Interobserver (semi-automated) | 0.525 |
| wavelet-LHL_firstorder_Minimum | Interobserver (semi-automated) | 0.526 |
| wavelet-LLL_glcm_ClusterProminence | NSCLC-Radiomics | 0.527 |
| original_firstorder_MeanAbsoluteDeviation | NSCLC-Radiomics | 0.529 |
| wavelet-LLH_glszm_SizeZoneNonUniformity | NSCLC-Radiomics | 0.530 |
| wavelet-LLH_gldm_DependenceNonUniformity | NSCLC-Radiomics | 0.534 |
| wavelet-LLL_glcm_Correlation | NSCLC-Radiomics | 0.535 |
| wavelet-HLH_glszm_SizeZoneNonUniformity | NSCLC-Radiomics | 0.535 |
| original_glrlm_RunEntropy | Interobserver (manual) | 0.537 |
| log-sigma-3-0-mm-3D_glrlm_RunLengthNonUniformity | NSCLC-Radiomics | 0.539 |
| original_glcm_Correlation | NSCLC-Radiomics | 0.540 |
| log-sigma-1-0-mm-3D_glszm_GrayLevelVariance | Interobserver (manual) | 0.540 |
| original_glszm_ZoneEntropy | NSCLC-Radiomics | 0.544 |
| log-sigma-2-0-mm-3D_glszm_GrayLevelNonUniformityNormalized | Interobserver (manual) | 0.545 |
| wavelet-LHL_glcm_JointAverage | Interobserver (semi-automated) | 0.545 |
| wavelet-LHL_glcm_SumAverage | Interobserver (semi-automated) | 0.545 |
| wavelet-HHL_firstorder_Kurtosis | Interobserver (semi-automated) | 0.545 |
| wavelet-LHL_firstorder_Minimum | Interobserver (manual) | 0.546 |
| original_firstorder_Kurtosis | Interobserver (manual) | 0.548 |
| original_firstorder_Entropy | Interobserver (manual) | 0.551 |
| wavelet-HHL_firstorder_Mean | Interobserver (semi-automated) | 0.551 |
| log-sigma-2-0-mm-3D_glrlm_RunLengthNonUniformity | NSCLC-Radiomics | 0.552 |
| original_gldm_DependenceEntropy | NSCLC-Radiomics | 0.552 |
| original_firstorder_RobustMeanAbsoluteDeviation | Interobserver (manual) | 0.553 |
| original_firstorder_Median | Interobserver (manual) | 0.553 |
| wavelet-LLH_gldm_SmallDependenceHighGrayLevelEmphasis | Interobserver (manual) | 0.553 |
| wavelet-HHH_glrlm_LongRunLowGrayLevelEmphasis | Interobserver (semi-automated) | 0.554 |
| wavelet-HLH_firstorder_Mean | NSCLC-Radiomics | 0.556 |
| original_gldm_SmallDependenceHighGrayLevelEmphasis | NSCLC-Radiomics | 0.556 |
| log-sigma-1-0-mm-3D_glszm_LowGrayLevelZoneEmphasis | Interobserver (manual) | 0.556 |
| original_firstorder_10Percentile | NSCLC-Radiomics | 0.556 |
| wavelet-LLL_firstorder_MeanAbsoluteDeviation | NSCLC-Radiomics | 0.557 |
| wavelet-LLL_gldm_DependenceEntropy | NSCLC-Radiomics | 0.557 |
| log-sigma-3-0-mm-3D_glszm_GrayLevelVariance | Interobserver (manual) | 0.557 |
| wavelet-LHL_glcm_SumAverage | Interobserver (manual) | 0.558 |
| wavelet-LHL_glcm_JointAverage | Interobserver (manual) | 0.558 |
| original_glcm_MCC | NSCLC-Radiomics | 0.558 |
| wavelet-LLH_glszm_LargeAreaLowGrayLevelEmphasis | Interobserver (manual) | 0.558 |
| wavelet-HHH_glszm_SmallAreaLowGrayLevelEmphasis | Interobserver (manual) | 0.559 |
| wavelet-HHH_gldm_LowGrayLevelEmphasis | Interobserver (manual) | 0.559 |
| wavelet-HHH_glrlm_LowGrayLevelRunEmphasis | Interobserver (manual) | 0.559 |
| wavelet-HHH_glrlm_ShortRunLowGrayLevelEmphasis | Interobserver (manual) | 0.559 |
| wavelet-HHH_glszm_LowGrayLevelZoneEmphasis | Interobserver (semi-automated) | 0.559 |
| wavelet-HHL_firstorder_Skewness | Interobserver (semi-automated) | 0.561 |
| wavelet-HHH_glszm_LowGrayLevelZoneEmphasis | Interobserver (manual) | 0.562 |
| wavelet-HLH_firstorder_Skewness | Interobserver (semi-automated) | 0.562 |
| wavelet-HHH_glrlm_LongRunLowGrayLevelEmphasis | Interobserver (manual) | 0.562 |
| wavelet-LLL_firstorder_10Percentile | NSCLC-Radiomics | 0.563 |
| wavelet-HLL_glcm_ClusterShade | Interobserver (manual) | 0.563 |
| log-sigma-1-0-mm-3D_glcm_MCC | Interobserver (semi-automated) | 0.563 |
| log-sigma-1-0-mm-3D_gldm_SmallDependenceHighGrayLevelEmphasis | Interobserver (semi-automated) | 0.564 |
| wavelet-HHH_gldm_SmallDependenceLowGrayLevelEmphasis | Interobserver (manual) | 0.564 |
| original_firstorder_InterquartileRange | Interobserver (manual) | 0.565 |
| wavelet-HHH_gldm_LowGrayLevelEmphasis | Interobserver (semi-automated) | 0.565 |
| wavelet-HHH_glszm_SmallAreaLowGrayLevelEmphasis | Interobserver (semi-automated) | 0.565 |
| wavelet-HHH_glrlm_LowGrayLevelRunEmphasis | Interobserver (semi-automated) | 0.566 |
| wavelet-LHL_glcm_MCC | Interobserver (manual) | 0.568 |
| wavelet-LLL_firstorder_RobustMeanAbsoluteDeviation | NSCLC-Radiomics | 0.568 |
| wavelet-HHH_glrlm_ShortRunLowGrayLevelEmphasis | Interobserver (semi-automated) | 0.570 |
| log-sigma-1-0-mm-3D_glrlm_ShortRunHighGrayLevelEmphasis | Interobserver (manual) | 0.571 |
| original_firstorder_Mean | Interobserver (manual) | 0.571 |
| log-sigma-1-0-mm-3D_glszm_SmallAreaHighGrayLevelEmphasis | Interobserver (manual) | 0.572 |
| wavelet-LLL_glcm_SumAverage | NSCLC-Radiomics | 0.573 |
| wavelet-LLL_glcm_JointAverage | NSCLC-Radiomics | 0.573 |
| wavelet-HHH_glcm_MCC | Interobserver (semi-automated) | 0.574 |
| original_glszm_GrayLevelVariance | Interobserver (manual) | 0.574 |
| wavelet-HLH_glcm_MCC | Interobserver (semi-automated) | 0.576 |
| original_firstorder_MeanAbsoluteDeviation | Interobserver (manual) | 0.579 |
| log-sigma-1-0-mm-3D_glrlm_LongRunLowGrayLevelEmphasis | Interobserver (semi-automated) | 0.581 |
| wavelet-LLL_glszm_LargeAreaLowGrayLevelEmphasis | Interobserver (semi-automated) | 0.581 |
| wavelet-LLL_firstorder_InterquartileRange | NSCLC-Radiomics | 0.582 |
| wavelet-LLH_glszm_SmallAreaHighGrayLevelEmphasis | Interobserver (manual) | 0.584 |
| original_shape_Sphericity | NSCLC-Radiomics | 0.584 |
| wavelet-LLL_gldm_SmallDependenceHighGrayLevelEmphasis | NSCLC-Radiomics | 0.585 |
| log-sigma-1-0-mm-3D_glszm_HighGrayLevelZoneEmphasis | Interobserver (manual) | 0.585 |
| wavelet-LLH_glszm_LargeAreaLowGrayLevelEmphasis | Interobserver (semi-automated) | 0.586 |
| original_glszm_SmallAreaHighGrayLevelEmphasis | NSCLC-Radiomics | 0.587 |
| original_firstorder_RootMeanSquared | Interobserver (manual) | 0.588 |
| wavelet-HHL_firstorder_RootMeanSquared | Interobserver (manual) | 0.589 |
| original_glcm_DifferenceVariance | Interobserver (manual) | 0.590 |
| original_glcm_Contrast | Interobserver (manual) | 0.591 |
| log-sigma-1-0-mm-3D_glszm_SmallAreaLowGrayLevelEmphasis | Interobserver (semi-automated) | 0.592 |
| original_glcm_JointEnergy | Interobserver (manual) | 0.592 |
| wavelet-HHL_glcm_MCC | Interobserver (manual) | 0.593 |
| log-sigma-2-0-mm-3D_glrlm_RunEntropy | NSCLC-Radiomics | 0.594 |
| wavelet-LHL_firstorder_Skewness | NSCLC-Radiomics | 0.594 |
| wavelet-LLH_firstorder_Minimum | Interobserver (manual) | 0.594 |
| original_glcm_Autocorrelation | NSCLC-Radiomics | 0.594 |
| original_firstorder_Kurtosis | Interobserver (semi-automated) | 0.595 |
| original_shape_Compactness1 | NSCLC-Radiomics | 0.596 |
| wavelet-LLL_glszm_SmallAreaHighGrayLevelEmphasis | NSCLC-Radiomics | 0.596 |
| wavelet-HLH_firstorder_RootMeanSquared | NSCLC-Radiomics | 0.597 |
| original_firstorder_10Percentile | Interobserver (manual) | 0.597 |
| log-sigma-1-0-mm-3D_glszm_LowGrayLevelZoneEmphasis | Interobserver (semi-automated) | 0.597 |
| wavelet-LLL_firstorder_Kurtosis | Interobserver (semi-automated) | 0.597 |
| wavelet-LHH_firstorder_Mean | NSCLC-Radiomics | 0.597 |
| wavelet-LLL_glszm_LargeAreaLowGrayLevelEmphasis | Interobserver (manual) | 0.598 |
| log-sigma-3-0-mm-3D_ngtdm_Coarseness | Interobserver (manual) | 0.599 |
| wavelet-HHL_gldm_SmallDependenceHighGrayLevelEmphasis | Interobserver (semi-automated) | 0.599 |

ICC - intraclass correlation coefficient; GLCM - gray level co-occurrence matrix, GLSZM - gray level size zone matrix, GLRLM - gray level run length matrix, NGTDM - neighbouring gray tone difference matrix, GLDM - gray level dependence matrix; Laplacian of Gaussian (LoG)
