# Supplementary material for: The impact of inter-observer variation in delineation on robustness of radiomics features in non-small cell lung cancer
Source: Sci Rep. 2022 Jul 27;12:12822. doi: 10.1038/s41598-022-16520-9 (PMC9329346; doi:10.1038/s41598-022-16520-9)
Supplement: Supplementary file 3 — Supplementary Information 3. [file 41598_2022_16520_MOESM3_ESM.docx]

**Supplementary Figure captions**

**Figure 1: Intraclass correlation coefficients (ICC) by feature type**

This figure separately considers the ICC values of radiomics features by different classes of features, as well as with the addition of Laplacian of Gaussian (LoG) and wavelet filters for the ‘NSCLC-Radiomics’, ‘Interobserver (manual)’ and ‘Interobserver (semi-automated)’ sets of contours, as well as an additional boxplot for a subset of patients from the ‘NSCLC-Radiomics’ dataset for whom the sets of contours have a Dice coefficient (DC) of greater than 0.6.

**Figure 2: Cumulative Intraclass correlation coefficient (ICC) plots**

The proportion of radiomics features with ICC less than 0.6, 0.8 and 0.9 are shown in this plot for the ‘NSCLC-Radiomics’, ‘Interobserver (manual)’ and ‘Interobserver (semi-automated)’ sets of contours, showing while less than 12.5% of features have an ICC less than 0.6, approximately 25% have an ICC less than 0.8 and approximately 50% have an ICC less than 0.9.

**Figure 3: Intraclass correlation coefficients (ICC) comparing radiomics features without and with resampling and resegmentation (IBSI compliant)**

This figure separately considers the ICC values of radiomics features by different classes of features, as well as with the addition of Laplacian of Gaussian (LoG) and wavelet filters for the ‘NSCLC-Radiomics’ sets of contours, comparing results for radiomics features that have not, with radiomics features that have been resampled and resegmented (IBSI compliant).

**Figure 4: Bland Altman plots**

The relationship between the Dice coefficients (DC) of pairs of contours within the ‘NSCLC-Radiomics’ dataset, the mean value of radiomic features and the absolute difference in the radiomics features for four features shown to be prognostic in a previously published model is presented here. While most sets of contours with high DC also show relatively low absolute differences in their radiomics feature result, there are a few outliers seen.

**Figure 5: Hazard ratios**

Hazard ratios and confidence intervals are given for overall survival for four radiomics features found to be prognostic in a previously published model, comparing PMCC to MAASTRO contours.
