# Supplementary material for: The impact of inter-observer variation in delineation on robustness of radiomics features in non-small cell lung cancer
Source: Sci Rep. 2022 Jul 27;12:12822. doi: 10.1038/s41598-022-16520-9 (PMC9329346; doi:10.1038/s41598-022-16520-9)
Supplement: Supplementary file 7 — Supplementary Information 7. [file 41598_2022_16520_MOESM7_ESM.pdf]

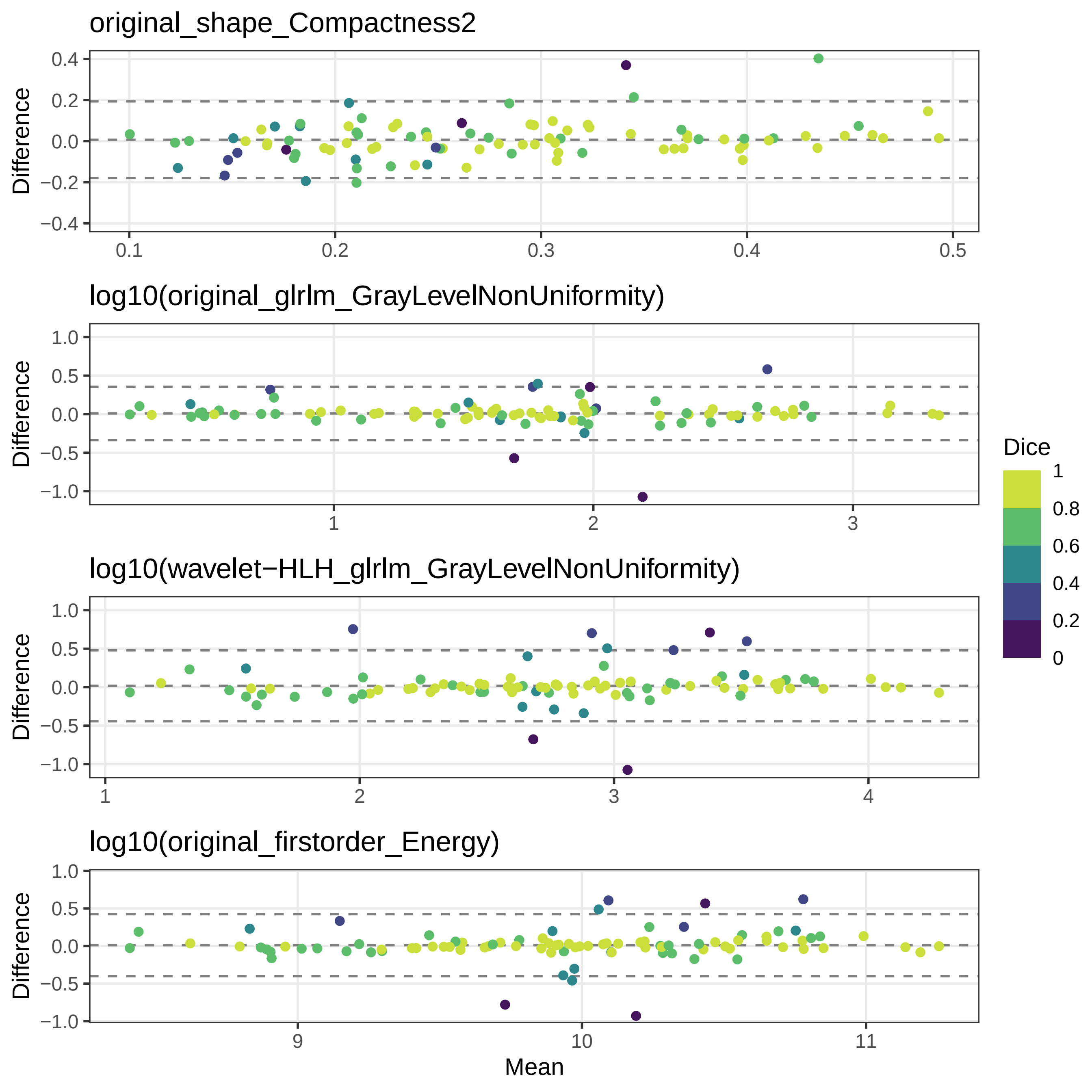

original_shape_Compactness2
log10(original_glrlm_GrayLevelNonUniformity)
log10(wavelet−HLH_glrlm_GrayLevelNonUniformity)
log10(original_firstorder_Energy)
Difference
Mean
Dice
1
0.8
0.6
0.4
0.2
0
