# Supplementary material for: The impact of inter-observer variation in delineation on robustness of radiomics features in non-small cell lung cancer
Source: Sci Rep. 2022 Jul 27;12:12822. doi: 10.1038/s41598-022-16520-9 (PMC9329346; doi:10.1038/s41598-022-16520-9)
Supplement: Supplementary file 8 — Supplementary Information 8. [file 41598_2022_16520_MOESM8_ESM.pdf]

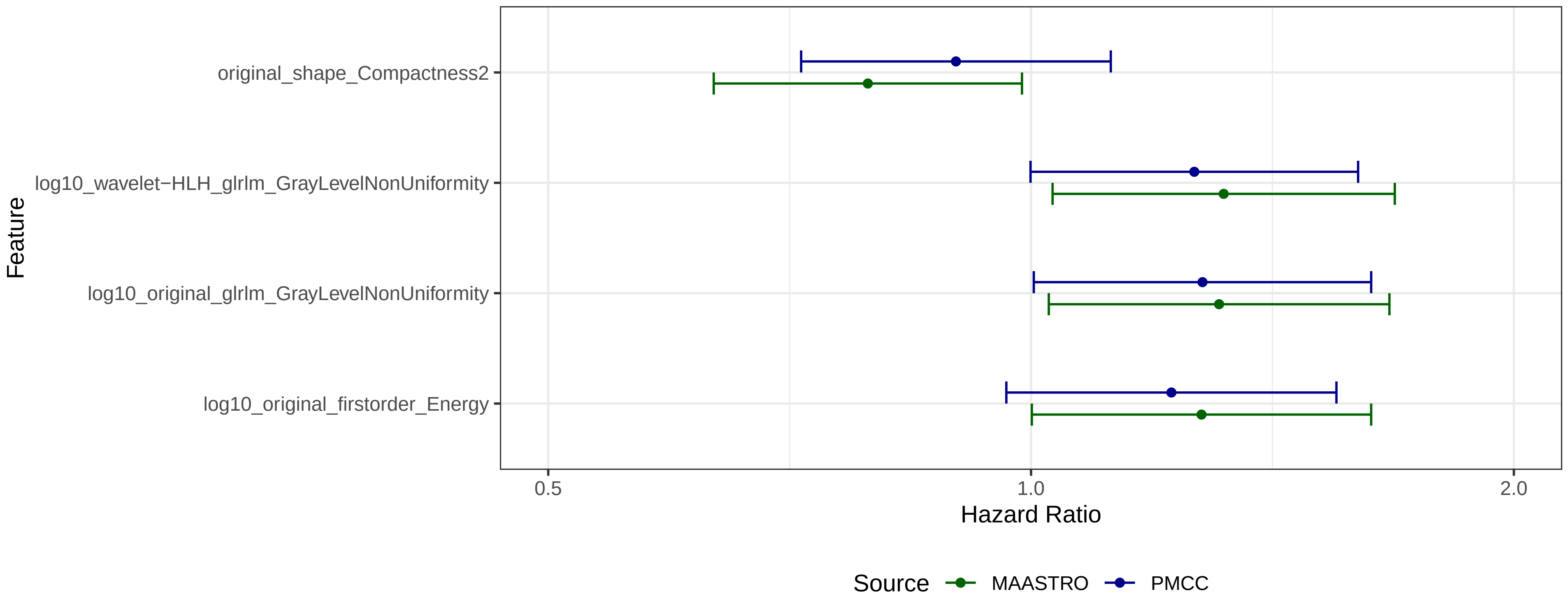

original_shape_Compactness2
log10_wavelet−HLH_glrlm_GrayLevelNonUniformity
log10_original_glrlm_GrayLevelNonUniformity
log10_original_firstorder_Energy
Feature
0.5
1.0
2.0
Hazard Ratio
Source
MAASTRO
PMCC
