# Supplementary material for: The impact of inter-observer variation in delineation on robustness of radiomics features in non-small cell lung cancer
Source: Sci Rep. 2022 Jul 27;12:12822. doi: 10.1038/s41598-022-16520-9 (PMC9329346; doi:10.1038/s41598-022-16520-9)
Supplement: Supplementary file 9 — Supplementary Information 9. [file 41598_2022_16520_MOESM9_ESM.docx]

# This is an example of settings that can be used as a starting point for analyzing CT data. This is only intended as a

# starting point and is not likely to be the optimal settings for your dataset. Some points in determining better values

# are added as comments where appropriate

# When adapting and using these settings for an analysis, be sure to add the PyRadiomics version used to allow you to

# easily recreate your extraction at a later timepoint:

# original_firstorder_energy

# original_shape_sphericity^3

# original_glrlm_greylevelnonuniformity

# wavelet-hlh_glrlm_greylevelnonuniformity

# ############################# Extracted using PyRadiomics version: <version> ######################################

imageType:

Original:

binWidth: 0.5

LoG:

binWidth: 10

sigma: [1.0, 2.0, 3.0]

Wavelet:

binWidth: 5

featureClass:

# redundant Compactness 1, Compactness 2 an Spherical Disproportion features are disabled by default, they can be

# enabled by specifying individual feature names (as is done for glcm) and including them in the list.

shape:

firstorder:

glrlm:

glszm:

gldm:

glcm:

ngtdm:

setting:

# Normalization:

# most likely not needed, CT gray values reflect absolute world values (HU) and should be comparable between scanners.

# If analyzing using different scanners / vendors, check if the extracted features are correlated to the scanner used.

# If so, consider enabling normalization by uncommenting settings below:

#normalize: true

#normalizeScale: 500 # This allows you to use more or less the same bin width.

# Resampling:

# Usual spacing for CT is often close to 1 or 2 mm, if very large slice thickness is used,

# increase the resampled spacing.

# On a side note: increasing the resampled spacing forces PyRadiomics to look at more coarse textures, which may or

# may not increase accuracy and stability of your extracted features.

#interpolator: 'sitkBSpline'

#resampledPixelSpacing: [1, 1, 1]

#padDistance: 10 # Extra padding for large sigma valued LoG filtered images

# Mask validation:

# correctMask and geometryTolerance are not needed, as both image and mask are resampled, if you expect very small

# masks, consider to enable a size constraint by uncommenting settings below:

#minimumROIDimensions: 2

#minimumROISize: 50

correctMask: false

# Image discretization:

# The ideal number of bins is somewhere in the order of 16-128 bins. A possible way to define a good binwidt is to

# extract firstorder:Range from the dataset to analyze, and choose a binwidth so, that range/binwidth remains approximately

# in this range of bins.

binWidth: 25

# first order specific settings:

voxelArrayShift: 1000 # Minimum value in HU is -1000, shift +1000 to prevent negative values from being squared.

# Misc:

# default label value. Labels can also be defined in the call to featureextractor.execute, as a commandline argument,

# or in a column "Label" in the input csv (batchprocessing)

label: 1
