# Supplementary material for: The impact of inter-observer variation in delineation on robustness of radiomics features in non-small cell lung cancer
Source: Sci Rep. 2022 Jul 27;12:12822. doi: 10.1038/s41598-022-16520-9 (PMC9329346; doi:10.1038/s41598-022-16520-9)
Supplement: Supplementary file 11 — Supplementary Information 11. [file 41598_2022_16520_MOESM11_ESM.docx]

**Supplementary Table 2: Features appearing in multiple datasets with intraclass correlation coefficient of less than 0.6**

| **Wavelet filter** | **Original (no filter)** | **LoG filter** |
| --- | --- | --- |
| wavelet-HHH_firstorder_Mean | original_firstorder_10Percentile | log-sigma-1-0-mm-3D_firstorder_90Percentile |
| wavelet-HHH_firstorder_RootMeanSquared | original_firstorder_InterquartileRange | log-sigma-1-0-mm-3D_glcm_MCC |
| wavelet-HHH_firstorder_Skewness | original_firstorder_Kurtosis | log-sigma-1-0-mm-3D_gldm_SmallDependenceHighGrayLevelEmphasis |
| wavelet-HHH_glcm_ClusterShade | original_firstorder_Mean | log-sigma-1-0-mm-3D_glszm_LowGrayLevelZoneEmphasis |
| wavelet-HHH_gldm_LowGrayLevelEmphasis | original_firstorder_MeanAbsoluteDeviation | log-sigma-1-0-mm-3D_glszm_SmallAreaLowGrayLevelEmphasis |
| wavelet-HHH_glrlm_LongRunLowGrayLevelEmphasis | original_firstorder_Median | log-sigma-2-0-mm-3D_firstorder_90Percentile |
| wavelet-HHH_glrlm_LowGrayLevelRunEmphasis | original_firstorder_Minimum | log-sigma-2-0-mm-3D_glszm_GrayLevelNonUniformityNormalized |
| wavelet-HHH_glrlm_ShortRunLowGrayLevelEmphasis | original_firstorder_RobustMeanAbsoluteDeviation | log-sigma-2-0-mm-3D_glszm_GrayLevelVariance |
| wavelet-HHH_glszm_LowGrayLevelZoneEmphasis | original_firstorder_RootMeanSquared | log-sigma-3-0-mm-3D_firstorder_90Percentile |
| wavelet-HHH_glszm_SmallAreaLowGrayLevelEmphasis | original_glcm_MCC | log-sigma-3-0-mm-3D_glszm_GrayLevelVariance |
| wavelet-HHL_firstorder_Mean | original_gldm_DependenceEntropy |  |
| wavelet-HHL_glcm_ClusterShade | original_glszm_GrayLevelVariance |  |
| wavelet-HLH_firstorder_Mean | original_glszm_ZoneEntropy |  |
| wavelet-HLH_firstorder_RootMeanSquared | original_ngtdm_Complexity |  |
| wavelet-HLH_firstorder_Skewness | original_shape_Compactness1 |  |
| wavelet-HLH_glcm_MCC | original_shape_Compactness2 |  |
| wavelet-LHH_firstorder_Mean | original_shape_Sphericity |  |
| wavelet-LHH_firstorder_RootMeanSquared |  |  |
| wavelet-LHH_firstorder_Skewness |  |  |
| wavelet-LHL_firstorder_Minimum |  |  |
| wavelet-LHL_glcm_Autocorrelation |  |  |
| wavelet-LHL_glcm_JointAverage |  |  |
| wavelet-LHL_glcm_SumAverage |  |  |
| wavelet-LHL_gldm_HighGrayLevelEmphasis |  |  |
| wavelet-LHL_gldm_LargeDependenceLowGrayLevelEmphasis |  |  |
| wavelet-LHL_gldm_SmallDependenceHighGrayLevelEmphasis |  |  |
| wavelet-LHL_glrlm_HighGrayLevelRunEmphasis |  |  |
| wavelet-LHL_glrlm_LongRunHighGrayLevelEmphasis |  |  |
| wavelet-LHL_glrlm_ShortRunHighGrayLevelEmphasis |  |  |
| wavelet-LHL_glszm_HighGrayLevelZoneEmphasis |  |  |
| wavelet-LHL_glszm_SmallAreaHighGrayLevelEmphasis |  |  |
| wavelet-LLH_glcm_Correlation |  |  |
| wavelet-LLH_gldm_LargeDependenceLowGrayLevelEmphasis |  |  |
| wavelet-LLH_glszm_LargeAreaLowGrayLevelEmphasis |  |  |
| wavelet-LLL_glcm_MCC |  |  |
| wavelet-LLL_gldm_LargeDependenceLowGrayLevelEmphasis |  |  |
| wavelet-LLL_glszm_LargeAreaLowGrayLevelEmphasis |  |  |

H – high pass filter; L – low pass filter; GLCM – gray level co-occurence matrix; GLRLM – gray level run length matrix; GLSZM – gray level size zone matrix; NGTDM – neighbouring gray tone difference matrix; GLDM – gray level dependence matrix; LoG – Laplacian of Gaussian
