# Supplementary material for: The impact of inter-observer variation in delineation on robustness of radiomics features in non-small cell lung cancer
Source: Sci Rep. 2022 Jul 27;12:12822. doi: 10.1038/s41598-022-16520-9 (PMC9329346; doi:10.1038/s41598-022-16520-9)
Supplement: Supplementary file 12 — Supplementary Information 12. [file 41598_2022_16520_MOESM12_ESM.docx]

**Supplementary Table 3: Frequency of features with intraclass correlation coefficient of less than 0.6**

| **Feature (with or without filter)** | **N** | **Feature (with or without filter)** | **N** |
| --- | --- | --- | --- |
| firstorder_Mean | 14 | glrlm_LongRunHighGrayLevelEmphasis | 2 |
| firstorder_Skewness | 13 | glrlm_RunLengthNonUniformity | 2 |
| glcm_MCC | 13 | glrlm_LowGrayLevelRunEmphasis | 2 |
| firstorder_RootMeanSquared | 11 | glszm_GrayLevelNonUniformity | 2 |
| gldm_LargeDependenceLowGrayLevelEmphasis | 10 | glrlm_ShortRunLowGrayLevelEmphasis | 2 |
| gldm_SmallDependenceHighGrayLevelEmphasis | 8 | glcm_SumSquares | 2 |
| firstorder_90Percentile | 8 | firstorder_Variance | 2 |
| glszm_GrayLevelVariance | 8 | gldm_LowGrayLevelEmphasis | 2 |
| glcm_ClusterShade | 8 | glcm_ClusterTendency | 2 |
| glszm_SizeZoneNonUniformity | 7 | glcm_JointEnergy | 2 |
| glszm_SmallAreaHighGrayLevelEmphasis | 6 | shape_Compactness2 | 2 |
| firstorder_Minimum | 6 | glrlm_GrayLevelVariance | 2 |
| firstorder_Kurtosis | 6 | glrlm_HighGrayLevelRunEmphasis | 2 |
| gldm_DependenceNonUniformity | 5 | gldm_HighGrayLevelEmphasis | 2 |
| glszm_LowGrayLevelZoneEmphasis | 4 | gldm_GrayLevelVariance | 2 |
| glszm_LargeAreaLowGrayLevelEmphasis | 4 | glcm_Contrast | 1 |
| firstorder_Median | 4 | gldm_SmallDependenceLowGrayLevelEmphasis | 1 |
| glcm_JointAverage | 4 | glcm_SumEntropy | 1 |
| glszm_SmallAreaLowGrayLevelEmphasis | 4 | glszm_SizeZoneNonUniformityNormalized | 1 |
| glcm_Correlation | 4 | ngtdm_Coarseness | 1 |
| glcm_SumAverage | 4 | glszm_SmallAreaEmphasis | 1 |
| ngtdm_Complexity | 3 | glcm_ClusterProminence | 1 |
| firstorder_InterquartileRange | 3 | firstorder_Entropy | 1 |
| firstorder_RobustMeanAbsoluteDeviation | 3 | glcm_DifferenceVariance | 1 |
| glrlm_LongRunLowGrayLevelEmphasis | 3 | firstorder_StandardDeviation | 1 |
| firstorder_MeanAbsoluteDeviation | 3 | glcm_MaximumProbability | 1 |
| glrlm_RunEntropy | 3 |  |  |
| shape_Compactness1 | 3 |  |  |
| glrlm_ShortRunHighGrayLevelEmphasis | 3 |  |  |
| firstorder_10Percentile | 3 |  |  |
| glszm_GrayLevelNonUniformityNormalized | 3 |  |  |
| glszm_ZoneEntropy | 3 |  |  |
| glcm_Autocorrelation | 3 |  |  |
| glszm_HighGrayLevelZoneEmphasis | 3 |  |  |
| gldm_DependenceEntropy | 3 |  |  |
| shape_Sphericity | 3 |  |  |
