# Supplementary material for: The impact of inter-observer variation in delineation on robustness of radiomics features in non-small cell lung cancer
Source: Sci Rep. 2022 Jul 27;12:12822. doi: 10.1038/s41598-022-16520-9 (PMC9329346; doi:10.1038/s41598-022-16520-9)
Supplement: Supplementary file 13 — Supplementary Information 13. [file 41598_2022_16520_MOESM13_ESM.docx]

**Supplementary Table 4: Features with intraclass correlation coefficient of less than 0.6**

| **Feature (resampled and re-segmented)** | **Dataset** | **ICC** |
| --- | --- | --- |
| wavelet-HHH_firstorder_Mean | NSCLC-Radiomics | 0.141 |
| original_glcm_ClusterShade | NSCLC-Radiomics | 0.224 |
| wavelet-LLL_glcm_ClusterShade | NSCLC-Radiomics | 0.228 |
| log-sigma-2-0-mm-3D_firstorder_90Percentile | NSCLC-Radiomics | 0.301 |
| original_firstorder_Median | NSCLC-Radiomics | 0.307 |
| log-sigma-2-0-mm-3D_gldm_DependenceNonUniformity | NSCLC-Radiomics | 0.311 |
| log-sigma-2-0-mm-3D_glszm_SizeZoneNonUniformity | NSCLC-Radiomics | 0.313 |
| wavelet-HHH_firstorder_RootMeanSquared | NSCLC-Radiomics | 0.341 |
| log-sigma-1-0-mm-3D_glszm_SizeZoneNonUniformity | NSCLC-Radiomics | 0.348 |
| log-sigma-3-0-mm-3D_gldm_DependenceNonUniformity | NSCLC-Radiomics | 0.349 |
| log-sigma-3-0-mm-3D_glszm_SizeZoneNonUniformity | NSCLC-Radiomics | 0.364 |
| log-sigma-1-0-mm-3D_firstorder_90Percentile | NSCLC-Radiomics | 0.367 |
| wavelet-LLL_glcm_ClusterTendency | NSCLC-Radiomics | 0.372 |
| original_glcm_ClusterTendency | NSCLC-Radiomics | 0.374 |
| wavelet-LHL_gldm_LargeDependenceLowGrayLevelEmphasis | NSCLC-Radiomics | 0.389 |
| log-sigma-3-0-mm-3D_firstorder_90Percentile | NSCLC-Radiomics | 0.390 |
| log-sigma-3-0-mm-3D_glrlm_RunEntropy | NSCLC-Radiomics | 0.400 |
| wavelet-LLL_glcm_SumSquares | NSCLC-Radiomics | 0.417 |
| original_glcm_SumSquares | NSCLC-Radiomics | 0.423 |
| wavelet-LLL_glszm_GrayLevelVariance | NSCLC-Radiomics | 0.429 |
| original_firstorder_Minimum | NSCLC-Radiomics | 0.431 |
| wavelet-LLL_firstorder_Median | NSCLC-Radiomics | 0.433 |
| wavelet-LLL_glrlm_GrayLevelVariance | NSCLC-Radiomics | 0.441 |
| wavelet-LLL_firstorder_Variance | NSCLC-Radiomics | 0.442 |
| wavelet-LLL_gldm_GrayLevelVariance | NSCLC-Radiomics | 0.442 |
| original_firstorder_Variance | NSCLC-Radiomics | 0.444 |
| original_gldm_GrayLevelVariance | NSCLC-Radiomics | 0.444 |
| original_glrlm_GrayLevelVariance | NSCLC-Radiomics | 0.444 |
| original_glszm_GrayLevelVariance | NSCLC-Radiomics | 0.446 |
| original_firstorder_Mean | NSCLC-Radiomics | 0.451 |
| wavelet-HHH_firstorder_Skewness | NSCLC-Radiomics | 0.470 |
| log-sigma-2-0-mm-3D_glszm_GrayLevelNonUniformity | NSCLC-Radiomics | 0.472 |
| wavelet-LLL_firstorder_Minimum | NSCLC-Radiomics | 0.472 |
| log-sigma-2-0-mm-3D_glszm_GrayLevelVariance | NSCLC-Radiomics | 0.477 |
| log-sigma-2-0-mm-3D_glszm_GrayLevelNonUniformityNormalized | NSCLC-Radiomics | 0.479 |
| original_firstorder_90Percentile | NSCLC-Radiomics | 0.491 |
| log-sigma-1-0-mm-3D_gldm_DependenceNonUniformity | NSCLC-Radiomics | 0.495 |
| wavelet-LLL_glszm_ZoneEntropy | NSCLC-Radiomics | 0.495 |
| original_firstorder_RootMeanSquared | NSCLC-Radiomics | 0.499 |
| original_firstorder_InterquartileRange | NSCLC-Radiomics | 0.499 |
| wavelet-LHH_gldm_LargeDependenceLowGrayLevelEmphasis | NSCLC-Radiomics | 0.499 |
| wavelet-LLL_firstorder_Mean | NSCLC-Radiomics | 0.501 |
| wavelet-LLL_glcm_MCC | NSCLC-Radiomics | 0.501 |
| wavelet-LLL_glszm_SizeZoneNonUniformity | NSCLC-Radiomics | 0.504 |
| original_firstorder_RobustMeanAbsoluteDeviation | NSCLC-Radiomics | 0.510 |
| log-sigma-1-0-mm-3D_gldm_LargeDependenceLowGrayLevelEmphasis | NSCLC-Radiomics | 0.513 |
| wavelet-LLL_gldm_DependenceNonUniformity | NSCLC-Radiomics | 0.515 |
| wavelet-LLL_firstorder_90Percentile | NSCLC-Radiomics | 0.515 |
| log-sigma-3-0-mm-3D_glszm_GrayLevelNonUniformityNormalized | NSCLC-Radiomics | 0.519 |
| original_glcm_JointAverage | NSCLC-Radiomics | 0.521 |
| original_glcm_SumAverage | NSCLC-Radiomics | 0.521 |
| wavelet-HLL_glszm_SizeZoneNonUniformity | NSCLC-Radiomics | 0.522 |
| original_firstorder_StandardDeviation | NSCLC-Radiomics | 0.525 |
| original_ngtdm_Complexity | NSCLC-Radiomics | 0.525 |
| log-sigma-3-0-mm-3D_glszm_GrayLevelNonUniformity | NSCLC-Radiomics | 0.525 |
| log-sigma-3-0-mm-3D_glszm_GrayLevelVariance | NSCLC-Radiomics | 0.525 |
| wavelet-LLL_glcm_ClusterProminence | NSCLC-Radiomics | 0.527 |
| original_firstorder_MeanAbsoluteDeviation | NSCLC-Radiomics | 0.530 |
| wavelet-LLH_glszm_SizeZoneNonUniformity | NSCLC-Radiomics | 0.530 |
| wavelet-LLH_gldm_DependenceNonUniformity | NSCLC-Radiomics | 0.535 |
| wavelet-LLL_glcm_Correlation | NSCLC-Radiomics | 0.536 |
| wavelet-HLH_glszm_SizeZoneNonUniformity | NSCLC-Radiomics | 0.536 |
| log-sigma-3-0-mm-3D_glrlm_RunLengthNonUniformity | NSCLC-Radiomics | 0.539 |
| original_glcm_Correlation | NSCLC-Radiomics | 0.540 |
| original_glszm_ZoneEntropy | NSCLC-Radiomics | 0.544 |
| log-sigma-2-0-mm-3D_glrlm_RunLengthNonUniformity | NSCLC-Radiomics | 0.552 |
| original_gldm_DependenceEntropy | NSCLC-Radiomics | 0.553 |
| wavelet-HLH_firstorder_Mean | NSCLC-Radiomics | 0.556 |
| original_gldm_SmallDependenceHighGrayLevelEmphasis | NSCLC-Radiomics | 0.557 |
| original_firstorder_10Percentile | NSCLC-Radiomics | 0.557 |
| wavelet-LLL_firstorder_MeanAbsoluteDeviation | NSCLC-Radiomics | 0.557 |
| wavelet-LLL_gldm_DependenceEntropy | NSCLC-Radiomics | 0.557 |
| original_glcm_MCC | NSCLC-Radiomics | 0.559 |
| wavelet-LLL_firstorder_10Percentile | NSCLC-Radiomics | 0.564 |
| wavelet-LLL_firstorder_RobustMeanAbsoluteDeviation | NSCLC-Radiomics | 0.568 |
| wavelet-LLL_glcm_JointAverage | NSCLC-Radiomics | 0.574 |
| wavelet-LLL_glcm_SumAverage | NSCLC-Radiomics | 0.574 |
| wavelet-LLL_firstorder_InterquartileRange | NSCLC-Radiomics | 0.583 |
| original_shape_Sphericity | NSCLC-Radiomics | 0.585 |
| wavelet-LLL_gldm_SmallDependenceHighGrayLevelEmphasis | NSCLC-Radiomics | 0.585 |
| original_glszm_SmallAreaHighGrayLevelEmphasis | NSCLC-Radiomics | 0.587 |
| log-sigma-2-0-mm-3D_glrlm_RunEntropy | NSCLC-Radiomics | 0.594 |
| wavelet-LHL_firstorder_Skewness | NSCLC-Radiomics | 0.595 |
| original_glcm_Autocorrelation | NSCLC-Radiomics | 0.595 |
| original_shape_Compactness1 | NSCLC-Radiomics | 0.597 |
| wavelet-LLL_glszm_SmallAreaHighGrayLevelEmphasis | NSCLC-Radiomics | 0.597 |
| wavelet-HLH_firstorder_RootMeanSquared | NSCLC-Radiomics | 0.597 |
| wavelet-LHH_firstorder_Mean | NSCLC-Radiomics | 0.598 |
