# Supplementary material for: The impact of inter-observer variation in delineation on robustness of radiomics features in non-small cell lung cancer
Source: Sci Rep. 2022 Jul 27;12:12822. doi: 10.1038/s41598-022-16520-9 (PMC9329346; doi:10.1038/s41598-022-16520-9)
Supplement: Supplementary file 14 — Supplementary Information 14. [file 41598_2022_16520_MOESM14_ESM.docx]

**Supplementary Table 5: Median value of radiomics features**

| **Feature** | **Dataset** | **n** | **Median** | **q1** | **q3** |
| --- | --- | --- | --- | --- | --- |
| original_shape_Compactness2 | NSCLC-Radiomics (MAASTRO contours) | 421 | 2.440859e-01 | 1.769565e-01 | 3.329410e-01 |
| original_shape_Compactness2 | NSCLC-Radiomics (MAASTRO contours) | 92 | 2.675467e-01 | 1.949851e-01 | 3.423644e-01 |
| original_shape_Compactness2 | NSCLC-Radiomics (PMCC contours) | 92 | 2.705796e-01 | 1.858538e-01 | 3.614564e-01 |
| original_firstorder_Energy | NSCLC-Radiomics (MAASTRO contours) | 421 | 1.185577e+10 | 2.723212e+09 | 3.220612e+10 |
| original_firstorder_Energy | NSCLC-Radiomics (MAASTRO contours) | 92 | 1.018404e+10 | 3.339355e+09 | 2.787321e+10 |
| original_firstorder_Energy | NSCLC-Radiomics (PMCC contours) | 92 | 9.284266e+09 | 3.338028e+09 | 2.716675e+10 |
| original_glrlm_GrayLevelNonUniformity | NSCLC-Radiomics (MAASTRO contours) | 421 | 5.984573e+01 | 1.121644e+01 | 2.115510e+02 |
| original_glrlm_GrayLevelNonUniformity | NSCLC-Radiomics (MAASTRO contours) | 92 | 5.987658e+01 | 1.834021e+01 | 2.172087e+02 |
| original_glrlm_GrayLevelNonUniformity | NSCLC-Radiomics (PMCC contours) | 92 | 5.574247e+01 | 1.851360e+01 | 1.801388e+02 |
| wavelet-HLH_glrlm_GrayLevelNonUniformity | NSCLC-Radiomics (MAASTRO contours) | 421 | 6.727472e+02 | 1.547799e+02 | 2.028959e+03 |
| wavelet-HLH_glrlm_GrayLevelNonUniformity | NSCLC-Radiomics (MAASTRO contours) | 92 | 5.870400e+02 | 2.048776e+02 | 1.669312e+03 |
| wavelet-HLH_glrlm_GrayLevelNonUniformity | NSCLC-Radiomics (PMCC contours) | 92 | 5.239516e+02 | 2.228170e+02 | 1.882242e+03 |
