# Supplementary material for: The impact of inter-observer variation in delineation on robustness of radiomics features in non-small cell lung cancer
Source: Sci Rep. 2022 Jul 27;12:12822. doi: 10.1038/s41598-022-16520-9 (PMC9329346; doi:10.1038/s41598-022-16520-9)
Supplement: Supplementary file 15 — Supplementary Information 15. [file 41598_2022_16520_MOESM15_ESM.docx]

**Supplementary Table 6: Hazard ratio for survival of radiomics features**

| **Source** | **Feature** | **Intraclass Correlation Coefficient** | **Hazard Ratio** | **p-value** | **95% confidence interval (low)** | **95% confidence interval (high)** |
| --- | --- | --- | --- | --- | --- | --- |
| MAASTRO | log10_original_glrlm_GrayLevelNonUniformity | 0.97 | 1.309924 | 0.030445 | 1.02581 | 1.672728 |
| PMCC | log10_original_glrlm_GrayLevelNonUniformity | 0.97 | 1.279055 | 0.046405 | 1.003929 | 1.629579 |
| MAASTRO | log10_wavelet-HLH_glrlm_GrayLevelNonUniformity | 0.95 | 1.318576 | 0.027356 | 1.031371 | 1.685759 |
| PMCC | log10_wavelet-HLH_glrlm_GrayLevelNonUniformity | 0.95 | 1.264138 | 0.050753 | 0.999232 | 1.599274 |
| MAASTRO | log10_original_firstorder_Energy | 0.91 | 1.277244 | 0.048952 | 1.001125 | 1.629519 |
| PMCC | log10_original_firstorder_Energy | 0.91 | 1.223193 | 0.095607 | 0.965149 | 1.550228 |
| MAASTRO | original_shape_Compactness2 | 0.61 | 0.791121 | 0.037953 | 0.63408 | 0.987056 |
| PMCC | original_shape_Compactness2 | 0.61 | 0.89781 | 0.341844 | 0.718873 | 1.121287 |
